# A LUHMES 3D dopaminergic neuronal model for neurotoxicity testing allowing long-term exposure and cellular resilience analysis

**DOI:** 10.1007/s00204-015-1637-z

**Published:** 2015-12-08

**Authors:** L. Smirnova, G. Harris, J. Delp, M. Valadares, D. Pamies, H. T. Hogberg, T. Waldmann, M. Leist, T. Hartung

**Affiliations:** 1 Center for Alternatives to Animal Testing (CAAT), Bloomberg School of Public Health, Johns Hopkins University, Baltimore, USA; 2 Center for Alternatives to Animal Testing (CAAT), Department of Biology, University of Konstanz, Konstanz, Germany

**Keywords:** 3D culture, Neurotoxicity, Resilience, microRNA, Rotenone

## Abstract

Several shortcomings of current Parkinson’s disease (PD) models limit progress in identification of environmental contributions to disease pathogenesis. The conditionally immortalized cell line LUHMES promises to make human dopaminergic neuronal cultures more easily available, but these cells are difficult to culture for extended periods of time. We overcame this problem by culturing them in 3D with minor medium modifications. The 3D neuronal aggregates allowed penetration by small molecules and sufficient oxygen and nutrient supply for survival of the innermost cells. Using confocal microscopy, gene expression, and flow cytometry, we characterized the 3D model and observed a highly reproducible differentiation process. Visualization and quantification of neurites in aggregates was achieved by adding 2 % red fluorescent protein-transfected LUHMES cells. The mitochondrial toxicants and established experimental PD agents, rotenone and MPP^+^, perturbed genes involved in one-carbon metabolism and transsulfuration pathways (*ASS1*, *CTH*, and *SHTM2*) as in 2D cultures. We showed, for the first time in LUHMES, down-regulation of *mir*-*7*, a miRNA known to target alpha-synuclein and to be involved in PD. This was observed as early as 12 h after rotenone exposure, when pro-apoptotic mir-16 and rotenone-sensitive mir-210 were not yet significantly perturbed. Finally, washout experiments demonstrated that withdrawal of rotenone led to counter-regulation of *mir*-*7* and *ASS1*, *CTH*, and *SHTM2* genes. This suggests a possible role of these genes in direct cellular response to the toxicant, and the model appears to be suitable to address the processes of resilience and recovery in neurotoxicology and Parkinson’s disease in future studies.

**Electronic supplementary material:**

The online version of this article (doi:10.1007/s00204-015-1637-z) contains supplementary material, which is available to authorized users.

## Introduction

Progress in the study of toxicant-induced dopaminergic neurodegeneration suffers from the shortcomings of available in vivo and in vitro models, since they usually do not progress in the same way human disease does. Moreover, important aspects such as endogenous counter-regulations and recovery are particularly difficult to address in vitro.

Although dopaminergic neurons correspond to <1 % of all neurons in the brain, they play a crucial role in movement, sensation of pleasure, motivation, reward, and drug addiction; are particularly sensitive to oxidative stress; and are involved in the second most common neurodegenerative disease—Parkinson’s disease (reviewed in Chinta and Andersen 2005). This neuronal cell type, therefore, is of particular interest for understanding the molecular mechanisms of PD—which are key to the development of preventive and disease-modifying therapies. Although genetic forms of PD associated with mutations in genes for *alpha*-*synuclein*, *PARKIN*, *PINK1*, *LRRK2* or *DJ*-*1* are well established (reviewed in Henchcliffe and Beal 2008), increasing evidence suggests a role for gene–environmental interactions contributing to the sporadic form of the disease, and gene regulatory networks are being unraveled (Kumar Singh et al. 2014; Krug et al. 2014; Fujita et al. 2014; Todorovic et al. 2014; Maertens et al. 2015; Lee and Cannon 2015; Rahnenführer and Leist 2015). Exposure to pesticides such as rotenone may be associated with increased risk of PD (Ascherio et al. 2006; Costello et al. 2009; Wang et al. 2011; Tanner et al. 2011). Mitochondrial dysfunctions (e.g., toxicant-induced mitochondrial complex I inhibition) are believed to be central in the pathophysiology of PD (reviewed in Franco-Iborra et al. 2015); however, it is not clear whether this is a primary or secondary event in PD pathogenesis. In addition, it is not clear yet why dopaminergic neurons are more vulnerable to mitochondrial complex I inhibition and degeneration. Thus, cellular responses to environmental stress and molecular perturbations upon toxicant insult leading to neurodegeneration need to be elucidated further.

There are a multitude of neuronal models for studying Parkinson’s disease and neurotoxicology. These include (a) cell lines, such as rat PC12 cell line (Greene and Tischler 1976; Grau and Greene 2012), SH-SY5Y neuroblastoma cell line (Constantinescu et al. 2007; Borland et al. 2008), and LUHMES cell line (Lotharius et al. 2005; Zhang et al. 2014; Stępkowski et al. 2015); (b) primary cell cultures (Lingor et al. 1999); and (c) embryonic stem cell (ESC)- or induced pluripotent stem cell (iPSC)-derived neurons (Srikanth and Young-Pearse 2014). Different types of neuronal models have strengths and limitations (Schlachetzki et al. 2012). The PC12 cell line, for instance, is easy to handle and relatively homogeneous, but is not of human origin—making it difficult to extrapolate interspecies differences in response to toxicant treatment. SH-SY5Y is a human cell line, fast dividing but difficult to differentiate into postmitotic neurons (Constantinescu et al. 2007) and has limitations as a cancer cell line because of its relatively unstable genome. Primary rat midbrain cell cultures were established to study PD (Lingor et al. 1999), but, again, with an obstacle of interspecies differences. Primary postmortem tissues, isolated from brains of patients with PD, more closely reflect the pathogenesis of the disease, but are difficult to obtain, are already affected by the disease, and, therefore, unsuitable for studying the dynamics of pathogenesis. Another limitation of primary cell cultures isolated from mesencephalon is low yield of biological material. In contrast to these primary or cancer tissue-originated cell models, the Lund human mesencephalic (LUHMES) cell line originates from healthy human 8-week-old embryonic mesencephalic tissue, immortalized by tetracycline-regulated *v*-*myc*-vector transfection, and can be rapidly and homogeneously differentiated to mature dopaminergic neurons by cultivation in the presence of tetracycline, cAMP, and GDNF (Lotharius et al. 2005; Scholz et al. 2011). Healthy donor origin of LUHMES makes these cells an attractive tool for studying the effects of environmental exposures leading to mitochondrial dysfunctions and/or to a PD-like phenotype. Human ESC and iPSC applications to study neurotoxicity and neurodegeneration are emerging and are very promising (Wheeler et al. 2015; Srikanth and Young-Pearse 2014), but the differentiation protocols are demanding, cost-prohibitive, and lengthy (up to 4/8 weeks of differentiation), resulting in heterogeneous neuronal populations. Protocols for enrichment in one cell type during differentiation, however, are evolving (Hu et al. 2015). Choosing one neural model over another depends on the hypotheses, specific aims, and study design. Although we believe iPSC-derived neural models will provide a broader range of options in future neurotoxicological studies, we also need models allowing easy access to homogenous cell populations for mechanistic studies. In this study, we favored the fast-differentiating and homogenous LUHMES cell line for the study of early cellular perturbations and cellular adaptation after toxicant exposure as a promising PD model.

More than 50 % of known miRNAs are expressed in the brain (Li and Jin 2010) where they posttranscriptionally regulate gene expression and play important roles in cellular homeostasis, metabolism, proliferation, differentiation, and apoptosis. Elimination of all miRNAs results in loss of the dopaminergic neurons in conditional knockout animal models and stem cells (Giraldez et al. 2005; Kim et al. 2007; Huang et al. 2010). Several miRNAs were shown to regulate function of dopaminergic neurons *mir*-*133b* (Kim et al. 2007), *mir*-*9* (Leucht et al. 2008), and *mir*-*132* (Yang et al. 2012). These and other dopaminergic neuron-specific miRNA were deficient in PD-affected midbrains (Kim et al. 2002; Lau and de Strooper 2010; Mouradian 2012). *mir*-*7*, which is expressed in nigral neurons in mice and humans, was shown to target α-synuclein and is down-regulated in MPP^+^ PD animal models (Junn et al. 2009). The neuroprotective role of *mir*-*7* against MPP^+^-induced neuronal death has been suggested (Fragkouli and Doxakis 2014; Choi et al. 2014). Thus, miRNAs may play an important role in cellular responses to toxicant exposure and PD development. An increasing number of studies are addressing whether miRNAs are involved in cellular responses to environmental stress (reviewed in Smirnova et al. 2012), and the role of miRNAs in neurotoxicity is being elucidated (Huang and Li 2009; Miranda et al. 2010; Saba et al. 2012; Tal and Tanguay 2012; Pallocca et al. 2013; Smirnova et al. 2014). Moreover, miRNAs play a significant role in mitochondrial function (Li et al. 2012), including pro-apoptotic *mir*-*15*, *mir*-*16*, and anti-apoptotic *mir*-*21*, *mir*-*17*-*92* cluster. ROS-responsive and hypoxia-related *mir*-*210* inhibits cell proliferation and represses the mitochondrial metabolism and respiration by targeting several elements of the TCA cycle (Chan et al. 2012). *mir*-*210*, together with *mir*-*195*, was shown to be regulated by rotenone exposure (Kim et al. 2013). Most importantly, there are mitochondria-enriched miRNAs, mito-miRs (Bandiera et al. 2013), which may be crucial in managing the mitochondrial response to toxicant-induced stress.

This work is based on our collaborative studies on the early responses of LUHMES cells to 1-methyl-4-phenylpyridinium (MPP^+^), the metabolite of 1-methyl-4-phenyl-1,2,3,6-tetrahydropyridine (MPTP), a common agent to experimentally induce PD (Krug et al. 2014), where perturbations and counter-regulations in the cells before mitochondrial dysfunction-initiated apoptosis were characterized. Based on data obtained from metabolomics and transcriptomics analysis, we proposed a network of toxicant (mitochondrial complex I inhibitor)-induced adaptations in human dopaminergic neurons before a tipping point is reached that allows execution of the apoptosis program. The next question is, are these early changes permanent, or can they be reversed after compound withdrawal? To experimentally test this, we need test systems that can be maintained for longer periods of time and interrogated for cellular perturbations, their counter-regulations, and ability to return to physiological conditions. LUHMES cells, which proved so promising in our earlier studies, do not allow such analysis in standard monolayer culture because differentiated cells tend to detach from the culture dish after about 9–12 days (depending on density, medium, and surface structuring).

3D cell cultures aiming to approximate organotypic cultures are rapidly emerging (Alépée et al. 2014; Hartung 2014), and they promise tissue-like cell density and cell/cell contacts. Therefore, for the first time, we have adapted the LUHMES cell culture to 3D using constant gyratory shaking. In the first step of the current study, we adapted the 2D protocol to generate a 3D dopaminergic neuronal model and found this prolonged survival of the differentiated cells. After the 3D protocol optimization and characterization steps, we demonstrated the suitability of the 3D model for neurotoxicity testing by using two model compounds, rotenone and MPP^+^. Finally, we analyzed the expression of miRNAs known to be involved in PD pathophysiology and mitochondrial function. We found that *mir*-*7* was sensitive to rotenone treatment and that its expression recovered after rotenone withdrawal, while MPP^+^ and rotenone-responsive genes *TYMS* and *MLF1IP*, identified previously in Krug et al. (2014), were further down-regulated with time. We demonstrated that the 3D LUHMES model is suitable for studying cellular responses after toxicant withdrawal and ultimately cellular resilience—which has previously not been possible in 2D because of short survival of these cultures.

## Materials and methods

### LUHMES maintenance and differentiation

Proliferation medium was prepared with Advanced DMEM/F12 (Gibco, Life Technologies) supplemented with 2 mM l-Glutamine (Sigma-Aldrich), 1× N2 (Gibco) and 0.04 μg/ml recombinant basic fibroblast growth factor (bFGF, R&D Systems). Differentiation medium was prepared with Advanced DMEM/F12 containing 2 mM l-Glutamine, 1× N2 supplement, 1 mM dibutyryl cAMP (Santa Cruz), 2 μg/ml tetracycline (Sigma-Aldrich), and 2 ng/ml recombinant human glial cell line-derived neurotrophic factor (GDNF, Gemini). For propagation and differentiation in monolayer, all flasks and plates were pre-coated with 50 μg/ml poly-l-ornithine (PLO) and 1 μg/ml fibronectin for 12 h prior to the experiment.

**Fig. 1:**
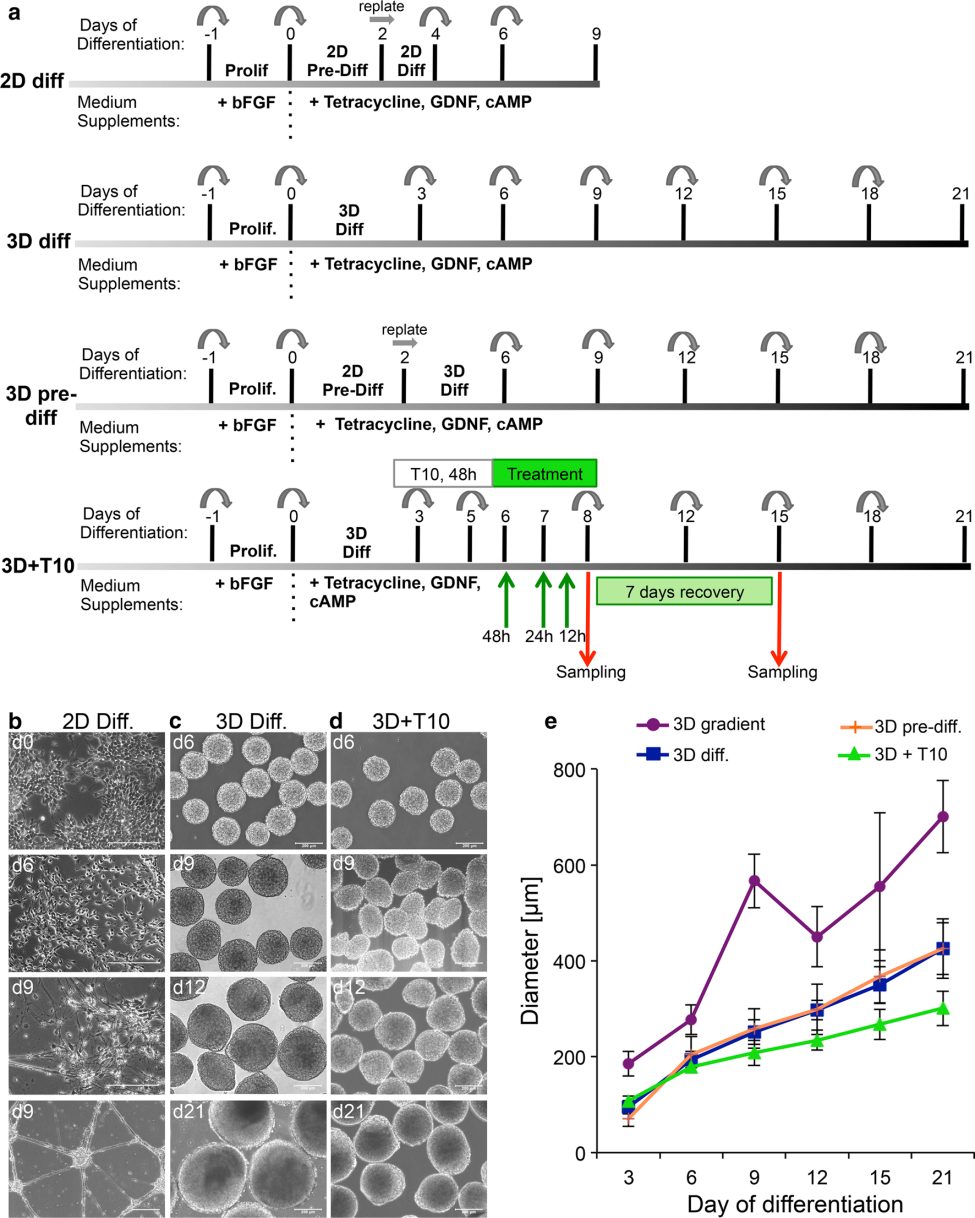
Adaptation and optimization of 3D LUHMES differentiation protocol. **a** The original 2D differentiation (2D diff protocol) was adapted for 3D culture (3D diff protocol) by subjecting the single-cell suspension to continuous gyratory shaking. Protocol 3D pre-diff involves a pre-differentiation step in 2D for 2 days, trypsinization, and subsequent cultivation in 3D. Further optimization involved adding the anti-proliferation compound taxol on day 3 for 48 h (10 nM, 48 h) to reduce cell proliferation (3D + T10 protocol). Toxic compound treatment took place between days 6 and 8 for 12, 24, and 48 h, reversely. Samples for toxicological end points were collected immediately after treatment on day 8 or on day 15 after washout of compounds and 7 days recovery. **b** Differentiation of LUHMES in monolayer. As differentiation advances, cells may detach from the surface (d9, last photograph). **c** Aggregate formation under continuous gyratory shaking (80 rpm). Note the increasing size of aggregates in course of differentiation. **d** Treatment of 3D cultures with anti-proliferation drug taxol (10 nM) for 48 h blocked the proliferation and slowed down aggregate growth. **e** Measurements of aggregate size during differentiation following different 3D protocols. At least 100 aggregates were measured per condition, per day, in at least three independent experiments, with exception of 3D pre-diff. and 3D gradient speed, where only one independent experiment was conducted. *Data* represent mean ± SEM, *n* ≥ 3 (independent experiments). *Scale bars* are 200 μm

Wild-type LUHMES (ATCC^®^ CRL_2927™) human neuronal precursor cells, as well as genetically modified LUHMES, ubiquitously expressing red or green fluorescent protein (RFP/GFP) were cultured as described previously (Scholz et al. 2011). RFP- and GFP-expressing cell lines were generated earlier as described in Schildknecht et al. (2013). Briefly, the conditionally immortalized cells (v-myc transgene expressing, controlled by a tet-off system) were maintained in proliferation medium in PLO–fibronectin pre-coated Nunclon™ (Nunc) flasks and passaged every 2–3 days. For differentiation in 2D (Fig. 1a, 2D diff protocol), cells were seeded in a pre-coated 175-cm^2^ flask (Nunc) in proliferation medium. After 24 h, medium was changed to differentiation medium. After 48 h in differentiation medium, cells were trypsinized using TripleE Express (Life Technologies) and seeded at a density of 5 × 10^5^ cells per 2 ml per well in pre-coated 6-well plates (Nunc™ Cell culture-treated). Medium was exchanged every second day.

To induce neuronal differentiation in 3D, LUHMES progenitors were trypsinized using TryplE Express, centrifuged, and resuspended in differentiation medium. Cells were seeded in 6-well plates (Falcon^®^) at 5 × 10^5^ cells/well in 2 ml of differentiation medium and placed on a gyratory shaker (ES-X, Kuhner shaker) at 80 rpm in a humidified incubator (37 °C, 10 % CO_2_). Medium was exchanged every third day by removing 1.2 ml and adding 1.5 ml of fresh medium (Fig. 1a, 3D diff protocol). Following protocol 3D + T10 (Fig. 1a) on day 3 of differentiation, aggregates were treated with 10 nM taxol (>97 % paclitaxel, T7191 Sigma-Aldrich), an anti-proliferation compound, for 48 h. On day 5, aggregates were washed directly in the well with washing medium (supplemented with l-glutamine, N2, and tetracycline), and differentiation medium was added to continue cellular differentiation. For co-cultures of wild-type LUHMES with RFP- or GFP-expressing cells, 1 × 10^4^ RFP or GFP cells were seeded with 5 × 10^5^ LUHMES-WT cells to comprise 2 % of the total cell population. Upon seeding, cells were differentiated as described above on a gyratory shaker at 80 rpm in a humidified incubator (37 °C, 10 % CO_2_).

### Chemical preparation, storage, and treatment

A 2 mM taxol stock was prepared in 100 % DMSO (D2650, Sigma-Aldrich), aliquoted, and stored at −20 °C. A 10 mM MPP^+^ iodite (>98 % HPLC powder, D048 Sigma-Aldrich) stock was prepared in sterile H_2_O, aliquoted, and stored at −20 °C protected from light. A 100 mM rotenone (>95 % powder, 84–79–4 Sigma-Aldrich) stock was prepared in 100 % DMSO, aliquoted, and stored at −20 °C protected from light. Once defrosted, aliquots were used immediately and discarded after use.

On day 3 of differentiation, aggregates were treated with taxol (10 nM, for 48 h) to reduce the number of proliferating cells. Test compounds (MPP^+^ and rotenone) were added to cultures on days 6, 7, or 7.5 for 48, 24, and 12 h, respectively. DMSO was used as a solvent control. End concentration of DMSO used in the cultures was ≤0.001 %, which did not have any effects on cell viability. Samples for toxicological end points were collected on day 8. To study cellular recovery, the toxicants were washed out on day 8. Cells were washed once with medium directly in the plate (supplemented with l-Glutamine, N2, and tetracycline) and plated in a new 6-well plate with fresh differentiation medium. Medium was exchanged on days 10 and 12. On day 15, aggregates were collected to measure end points relevant to cellular recovery from the effects of rotenone or MPP^+^ exposure.

### Cell viability

Cell viability (mitochondria activity) was analyzed using the resazurin reduction assay. A 1 mg/ml resazurin sodium salt (Sigma-Aldrich) stock was prepared in PBS. 200 μl of 1 mg/ml stock was added to each well (2 ml medium), and plates were kept on a gyratory shaker at 80 rpm in a humidified incubator (37 °C, 10 % CO_2_) for 1.5 h. 100 μl of samples was transferred from each well in triplicates into 96-well plates, and fluorescence was measured in a fluorescence plate reader (530 nm excitation/590 nm emission). Differentiation medium was incubated with resazurin in parallel as a blank control. Cell viability (mitochondria activity) was calculated as % of fluorescence intensity relative to solvent-treated controls after subtracting blanks in three biological replicates.

Cytotoxicity (membrane integrity) was analyzed using the LDH release assay (Promega). As a positive control, aggregates were treated with 1 % Triton X100. 50 μl of medium from each well was transferred to a 96-well plate. 50 μl of LDH substrate was added to each well. The plate was incubated for 30 min at room temperature in the dark. Differentiation medium was incubated with substrate in parallel as a blank control. The reaction was stopped with 50 μl stop solution. Absorbance was recorded at 490 nm. After subtracting blanks, cytotoxicity (%) was determined by normalization of ODs from the test sample to positive and solvent-treated controls.

### Mitochondrial activity

Mitochondrial activity after rotenone treatment was measured using the red fluorescent dye Mitotracker^®^ Red CMXRos (Life Technologies) following the manufacturer’s instructions. Briefly, after 48-h rotenone exposure, aggregates were transferred into 24-well plates and incubated with 1 μM Mitotracker^®^ Red on a gyratory shaker at 80 rpm at 37 °C, 10 % CO_2_ for 30 min. Aggregates were washed twice with PBS and fixed with 4 % PFA for 20 min at 4 °C. Fixed aggregates were washed twice in PBS and mounted on slides for fluorescence imaging (excitation 579 nm/emission 599 nm). In every independent experiment, at least 10 single aggregates were imaged per condition, and the mean gray value (sum of the gray values of all the pixels in the area divided by the number of pixels in the area) per aggregate was quantified using the open-source ImageJ software (http://imagej.nih.gov/ij/) and normalized to solvent-treated controls. Average of normalized mean gray values ± SEM was calculated from at least three biological replicates. Differences in treated and control samples were analyzed for statistical significance using Kruskal–Wallis test followed by Dunn’s Multiple comparison test. *p* value is denoted on graphs by ***.

### Size measurements

Aggregates were cultured as described above, and phase-contrast microscopic images were taken on days 3, 6, 9, 12, 15, and 21 of differentiation. The diameter of 20–50 aggregates was measured on each day using SPOT software 5.0 (Diagnostic Instruments, Inc.). Experiments were repeated at least three times.

### Flow cytometry

2D and 3D differentiated, as well as undifferentiated, LUHMES cells were trypsinized directly in the plate on the shaker with TryplE Express containing 4 units/ml DNAse at 37 °C for 30 min. After 30 min, aggregates were homogenized by aspiration using a 1-ml syringe with 26G3/8 needle. Single-cell suspensions in 2D were obtained by pipetting up and down several times. The previously described protocol (Smirnova et al. 2015a) was followed for further steps. Briefly, cells were fixed with 2 % PFA and stained with Alexa Flour 647 mouse antihuman Ki-67 antibody (1:20, clone B56, BD Pharmingen™) at 4 °C for 1 h in PBS/1 %BSA/0.15 % saponin/10 % goat serum. Ki-67 expression was quantified using a FACSCalibur flow cytometer (BD). Unstained cells, as well as cells stained with Alexa Flour 647 mouse IgG1 *κ* Isotype control, were used to set the gates for Ki-67-negative cells.

### Immunocytochemistry and confocal imaging

For immunostaining, aggregates were collected on days 6, 12, 15, or 21 of differentiation, and immunocytochemistry was performed as previously described (Smirnova et al. 2015a) with some adaptations for 3D cultures. Briefly, aggregates were fixed with 4 % PFA (20 min, 4 °C) and blocked with blocking solution [10 % goat serum (Sigma), 1 % BSA (Sigma), 0.15 % saponin in PBS] for at least 1 h on a shaker at 4 °C. Subsequently, aggregates were incubated with primary antibodies diluted in blocking solution for 48 h at 4 °C on a shaker. The following antibodies were used; they are as follows: rabbit antihuman neurofilament (NF200, 1:200, Sigma); mouse antihuman MAP2 (1:100, Sigma); mouse antihuman NeuN (1:100, Millipore); mouse antihuman synaptophysin (1:200, Millipore); rabbit antihuman Ki-67 (1:100, Santa Cruz). As negative control, aggregates were incubated with blocking solution. Then, aggregates were washed twice and incubated for 24 h at 4 °C on a shaker in the dark with secondary antibodies (goat antimouse IgG Alexa Fluor^®^ 488 and goat antirabbit IgG Alexa Fluor^®^ 568, 1:500, Life Technologies). After 24 h, aggregates were washed, and nuclei were stained with Hoechst 33342 (1 μg/ml, Invitrogen, Molecular Probes) for at least 1 h at RT. Aggregates were then mounted using mounting medium (Immu-Mount™, Thermo Scientific) on glass slides (Fisherbrand^®^ Thermo Scientific) and analyzed using the Zeiss LSM 510 Confocal III confocal microscope (Zeiss) and ZEN Imaging software (Zeiss).

For dye penetration assays, LUHMES ubiquitously expressing GFP were used. GFP-LUHMES were differentiated in 3D for 12 days following 3D diff protocol (Fig. 1a). On day 12 of differentiation, Hoechst 33342 (1 μg/ml) was added to the cultures for 5, 15, 30, 60 min, or 6 h. Aggregates were then fixed with 4 % PFA, incubated with optical clearing solution (Sca*l*eA2: 4 M urea, 10 % wt/vol glycerol, 0.1 % wt/vol Triton-X-100, pH 7.7 (Hama et al. 2011) for further 48 h at 4 °C on a shaker and mounted on the glass slides. Hoechst 33342 penetration through the aggregates was analyzed using Zeiss LSM 510 confocal III microscope.

### Neurite integrity quantification

To assay neurite integrity, RFP-expressing LUHMES were mixed with wild-type LUHMES in a ratio 1:49. Cells were differentiated following 3D + T10 protocol. On day 6, cells were exposed to 0.1 μM rotenone or DMSO as control. After 48-h exposure, aggregates were collected, fixed with 4 % PFA, and nuclei were stained with 1 μg/ml Hoechst 33342. Then, aggregates were incubated with Sca*l*eA2 solution for further 48 h at 4 °C on a shaker and mounted on glass slides for imaging by confocal microscopy. The hyperstack images were analyzed using the software KNIME with the Image Processing plugin. The program provides algorithms and means for image (pre) processing, filtering, segmentation, and feature calculation. The provided algorithms are well known and common image analysis techniques that can be combined to fit arbitrary analysis problems. In order to quantify the neurites, edge detection was applied to the images to highlight the neurites. Then, the neurite areas as well as the cell bodies were segmented. After cell bodies were excluded from the analysis, the area of the identified neurites for every slice of the hyperstack was calculated and normalized to the number of red cells in the aggregate. Lastly, the median neurite area within the hyperstack was calculated in three biological replicates (mean ± SEM). Differences in rotenone-treated and DMSO-treated samples were analyzed for statistical significance using the Mann–Whitney test; *p* value <0.01 is denoted on the graphs by **.

### Apoptosis assays

Two assays were used to estimate the level of apoptosis during 3D differentiation (3D diff protocol). The number of apoptotic/necrotic cells was quantified using PE Annexin V Apoptosis Detection kit I (BD Pharmingen™) by flow cytometry and visualized using the Cell Event^®^ Caspase-3/7 Green Detection Reagent (Life technologies) by confocal fluorescent microscopy. For Annexin V-PE/7AAD staining, aggregates were trypsinized for 30 min with TryplE Express, washed once with PBS, and resuspended in 1× Annexin V binding buffer. 4 × 10^5^ cells were stained in 100 μl with 5 μl Annexin V-PE and 5 μl 7-AAD for 15 min at room temperature protected from light. Unstained cells and cells stained either with Annexin V or 7-AAD were used to set the gates. Cells treated with 0.5 μM rotenone were used as positive control. The percentage of Annexin V-positive cells, 7-AAD-positive cells, and double-positive cells were quantified using a FACSCalibur flow cytometer (BD). For caspase 3/7 staining, upon addition of the Cell Event reagent and 1 μg/ml Hoechst 33342, cells were incubated at 37 °C for 60 min, washed once with PBS, and fixed with 4 % PFA for 20 min at 4 °C on a shaker. Fixed aggregates were washed twice with PBS and mounted on slides for fluorescence imaging using Zeiss LSM 510 confocal III microscope. Undifferentiated LUHMES were used as negative control in both experiments.

### RNA extraction, Reverse Transcription, and Real-Time PCR

Total RNA was extracted using either TRIzol^®^ Reagent (Life Technologies) or the miRVANA miRNA isolation kit (Ambion, Life Technologies) following the manufacturer’s instructions. RNA integrity was measured using the Nanodrop 2000 (TermoScientific) UV–Vis Spectrophotometer (260 nm).

Equal amounts of purified RNA (500 ng) were reverse transcribed to cDNA using random hexamer primers (Promega) and M-MLV reverse transcriptase Kit (Promega) following the manufacturer’s instructions. A DNAse treatment step was included in cDNA synthesis to ensure the elimination of DNA traces. cDNA was diluted 1:5, and qRT-PCR was performed. Expression of neuronal markers during LUHMES differentiation was analyzed using TaqMan gene expression assay (Life Technologies) and TaqMan advance Master Mix (Life Technologies) according to the manufacturer’s protocols. Expression of genes perturbed by toxicant treatment was analyzed using Fast SYBR Green master mix (Life Technologies) and primers listed in Supplementary Table S1. 18S and GAPDH were used as housekeeping genes for TaqMan gene expression and SYBR Green PCRs, respectively. All RT-PCRs were performed in duplicates on Fast Applied Biosystems 7500 System (Life Technologies) with the following thermal cycling parameters: SYBR^®^ Green RT-PCR (95 °C for 20 s, followed by 40 cycles of 3 s at 95 °C and 30 s 60 °C); a melting curve step was included in SYBR Green reactions (95 °C for 15 s, 60 °C for 1 min, 95 °C for 15 s, and 60 °C for 15 s); TaqMan gene expression assay (95 °C for 20 s, followed by 40 cycles of 3 s at 95 °C and 30 s 60 °C).

For miRNA amplification, short stem-loop cDNA libraries from individual miRNAs were generated using TaqMan^®^ microRNA assays and TaqMan^®^ microRNA reverse transcription kits (Life Technologies). Up to six miRNAs were multiplexed in one reaction. Quantitative real-time PCR on miRNAs was performed using the TaqMan^®^ microRNA assay kit in combination with TaqMan^®^ FAST advanced PCR master mix, (Life Technologies) with the following thermal cycling parameters: 95 °C for 20 s, followed by 40 cycles of 3 s at 95 °C and 30 s 60 °C. Expression of individual miRNAs was normalized to *RNU44* expression and was shown relative to expression in solvent-treated LUHMES.

The relative mRNA and miRNA expression was quantified using the comparative CT (2^−∆∆CT^) method (Schmittgen and Livak 2008). Data collected from three to four independent experiments were calculated as average log_2_-fold change in independent biological replicates ± SEM. Differences in treated and control samples were analyzed for statistical significance using one-way ANOVA test followed by Dunnett’s post hoc test. *p* value <0.05 is denoted on graphs by *, *p* < 0.01 by **, and *p* < 0.001 by ***, respectively.

## Results

### Protocol adaptation and optimization for LUHMES differentiation in 3D

Differentiation of LUHMES cells in monolayer culture (Fig. 1a, b, 2D diff protocol) was well established and extensively characterized earlier (Scholz et al. 2011; Schildknecht et al. 2013), and the described changes in morphology, proliferation (Ki-67), and differentiation (Map2 and NF200) have been reproduced here (Supplementary Figure S1a). However, LUHMES cells differentiated in monolayer have limited life span. With increasing culture age, the interaction of the large neurite network with the extracellular matrix (plate coating) weakens, and the network either contracts into ganglion-like structures or fully detaches from the plate (Fig. 1b, last panel, d9). The brief survival of the differentiated cultures in 2D (which allows acute toxicity studies) is an obstacle for long-term, low-dose toxicological studies, as well as for cellular adaptation and resilience studies after toxicological stress. Therefore, we modified and adapted the LUHMES differentiation protocol for 3D. The 3D LUHMES model was prepared using the gyratory shaking technique as established for 3D rat primary aggregating brain cell cultures (Honegger and Monnet-Tschudi 2001; van Vliet et al. 2008) and iPSC microphysiological systems (Hogberg et al. 2013) with few modifications (Fig. 1a, c, 3D diff protocol).

First, the size of aggregates was monitored during differentiation. By adjusting initial cell number and shaker speed, we were able to control aggregate size (Fig. 1e, 3D diff). The cultivation of the aggregates from day 0 of differentiation under a constant shaking speed of 80 rpm allowed us to keep the size of aggregates within 300–425 μm in diameter through 21 days of differentiation, while under a gradually increasing speed from 68 to 80 rpm during the first 5 days, as originally established for rat primary cultures (Honegger and Monnet-Tschudi 2001), LUHMES aggregates reached 700 μm in diameter (Fig. 1e, 3D gradient).

**Fig. 2:**
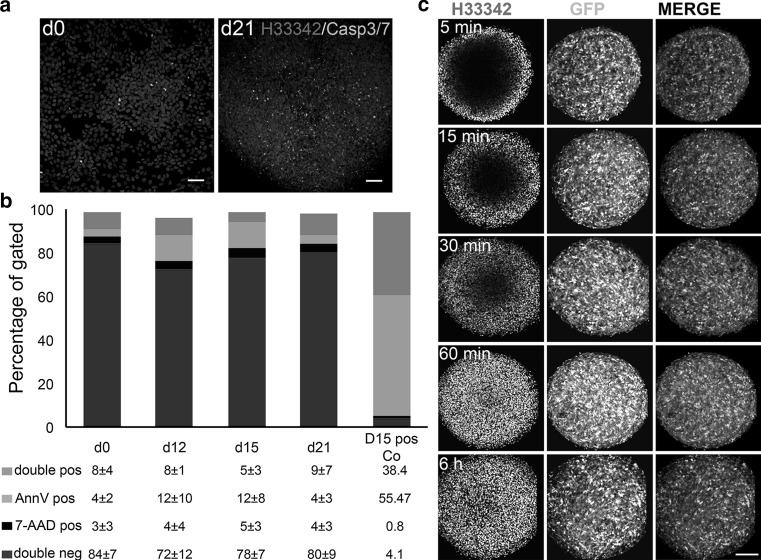
Quantification of apoptosis and necrosis level in 3D LUHMES model. **a** Caspase 3/7 activation (*green* nuclei) as an early apoptotic marker was visualized using fluorescent microscopy in combination with Hoechst 33342 staining of nuclei (*blue*) in undifferentiated LUHMES monolayer cultures (d0) and 21 days after induction of differentiation in 3D. *Scale bars* are 50 μm. **b** Annexin V/7-AAD-positive cells were quantified using flow cytometry on day 0 (as negative control) and days 12, 15, and 21 following 3D diff. protocol for differentiation. Aggregates exposed to 0.5 μM rotenone for 48 h and after 7 days recovery were used as a positive control (*last panel*). Annexin V-positive cells are in early apoptosis; double stained for Annexin V and 7AAD cells are in later apoptotic phases, while 7-AAD-positive cells represent a population of necrotic cells. Data are shown as mean ± SEM, *n* ≥ 3 (independent experiments) **c** Penetration assay with Hoechst 33342: 12-day-old aggregates of GFP-expressing LUHMES were differentiated according to 3D diff protocol and were stained for increasing time intervals with Hoechst 33342. Confocal optical slices through the center of the aggregates are shown to demonstrate time-dependent penetration of Hoechst 33342 through the aggregates. Compare GFP expression (*green*) at all time points in the center of aggregates with the absence of Hoechst 33342 staining (*blue*) after 5, 15, and 30 min of incubation and penetration of Hoechst 33342 to the middle after 1 and 6 h of incubation. No apoptotic nuclei are visible in the center of aggregates (60 min and 6 h). *Scale bar* is 100 μm (color figure online)

Second, in order to test sufficient oxygen and nutrient supply, early apoptosis (Annexin V and caspase 3/7-positive cells) and necrosis (7-AAD-positive cells) were monitored by flow cytometry and fluorescence microscopy. Although a low percentage of caspase 3/7-positive cells were detectable on day 21 of differentiation (Fig. 2a), caspase 3/7-positive cells were distributed equally throughout the aggregates without any visible accumulation in the middle of the aggregates. No increase in Annexin V-positive cells was observed during the 21 days of differentiation in 3D (Fig. 2b). The percentage of Annexin V- and 7AAD-positive cells in the 3D cultures was comparable to those in monolayer undifferentiated LUHMES cultures, which were subjected to the same preparation procedure—both cultures were trypsinized for 30 min prior to Annexin V/7-AAD staining.

Third, we investigated compound penetration by staining the live, 12-day-old aggregates with DNA-binding blue fluorescent dye, Hoechst 33342 trihydrochloride, MW = 616 g/mol (Invitrogen) for 5, 15, 30, 60 min, and 6 h. For this experiment, LUHMES ubiquitously expressing GFP were used (Schildknecht et al. 2013). Hoechst 33342 dye penetration throughout the aggregates was advancing with increasing incubation time. Hoechst 33342 reached the middle of the aggregates after 1 h of treatment (Fig. 2c, Supplementary Figure S1b). This experiment ensured sufficient penetration of necessary small molecule factors for differentiation and nutrients, as well as toxicants. In addition, no visible apoptotic nuclear fragmentation accumulated in the middle of aggregates (Fig. 2c, 1 and 6 h). Thus, 3D cultures could be kept at least twice as long in culture than their 2D counterparts.

**Fig. 3:**
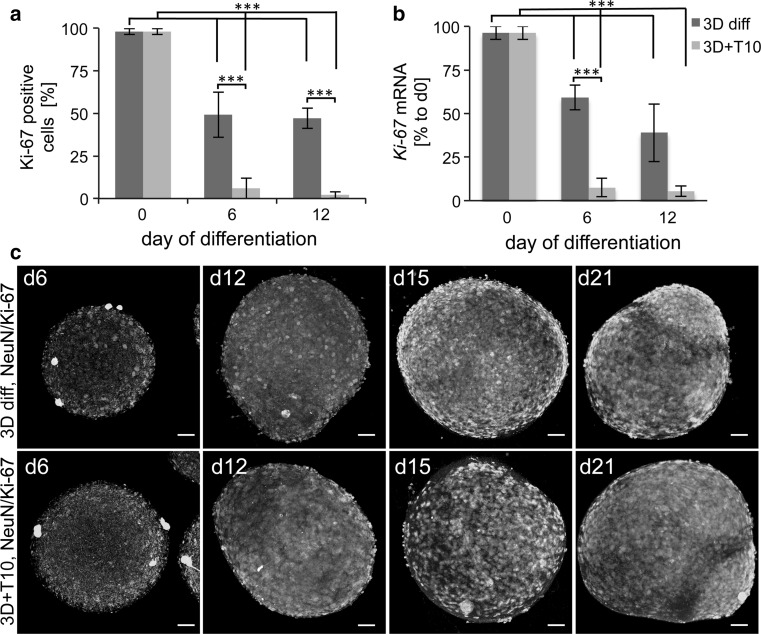
Estimation of proliferation rate within the aggregates. **a** Percentage of Ki-67-positive cells on days 6 and 12 of differentiation with or without taxol in comparison with undifferentiated LUHMES (d0). The number of Ki-67-positive cells was measured using Alexa Flour 647-conjugated anti-Ki-67 antibody by flow cytometry. *Data* represent mean ± SD, *n* ≥ 3 (independent experiments). **b** *Ki*-*67* gene expression on days 0, 6, and 12 of differentiation in 3D diff and 3D + T10 cultures. Data are normalized to *Ki*-*67* expression on d0 and represent mean ± SEM, *n* ≥ 3 (independent experiments). **c** Immunostainings of 3D diff and 3D + T10 aggregates with antibody against KI-67 showing prolonged presence of Ki67-positive cells (*red*) in 3D diff cultures in comparison with 3D + T10 aggregates. The aggregates were co-stained with postmitotic neuronal marker NeuN (*green*). The nuclei were visualized with Hoechst 33342 staining. *Scale bars* are 50 μm. The aggregates were fixed on glass slides and covered with coverslips for confocal imaging, which explains the larger size of the aggregates in comparison with Fig. 1, where floating aggregates were imaged (color figure online)

Finally, withdrawal of FGF and the addition of tetracycline, cAMP, and GDNF should rapidly induce exit from the cell cycle and differentiation to postmitotic mature neurons. We suggest that the observed continuous increase in the size of aggregates during differentiation could be due to a prolonged proliferation in 3D differentiating cultures. Higher cell density and increased cell-to-cell interactions may stimulate signaling between the cells within the aggregates that impedes exit from the cell cycle. Therefore, we quantified the expression of Ki-67, a proliferation marker, in undifferentiated cells as well as in 2D and 3D cultures. As expected, undifferentiated LUHMES were 98 ± 2 %-positive for Ki-67. Induction of differentiation in 2D reduced the expression of Ki-67 to 16 % by day 6, while in 3D cultures 49 ± 13 % of the cells were still Ki-67-positive on day 6 and 47 ± 12 % on day 12 (Fig. 3a, Supplementary Figure S1c). Therefore, we optimized the 3D diff protocol further to accelerate the exit from cell cycle in 3D and induce homogeneous differentiation. In the first step, we evaluated whether pre-differentiation in 2D for 48 h before 3D differentiation (Fig. 1a, 3D pre-diff protocol) would decrease proliferation. No differences in the size of aggregates (Fig. 1e, 3D pre-diff), as well as no change in Ki-67 expression (data not shown), were observed, compared to the 3D diff protocol; this protocol, therefore, was not followed further. In the second step, we tested whether increasing the tetracycline concentration would reduce the proliferation rate. LUHMES were differentiated according to the 3D diff protocol in the presence of 2, 4, and 10 μg/ml tetracycline. Although the highest tetracycline concentration reduced the proportion of proliferating Ki-67 cells (Supplementary Figure S1d), it appeared to be cytotoxic for the cultures (observation based on aggregate morphology, data not shown). Next, we applied treatment with the mitotic inhibitor taxol (also known as paclitaxel). The supplementation of neural differentiation media with anti-proliferation drugs, such as cytosine arabinofuranoside (AraC), is common and broadly used in primary neuronal cultures to block the proliferation of neuroprogenitors and astroglia without affecting postmitotic neurons (Gerhardt et al. 2001; Volbracht et al. 2006) After optimization experiments, 10 nM taxol for 48 h, from days 3 to 5 of differentiation, was chosen as a treatment scheme (Fig. 1a, 3D + T10 protocol). Treatment with taxol led to a reduction in aggregate size (250–300 μm on average, Fig. 1d, e, 3D + T10) and significant decreased in Ki-67-positive cells to 6 ± 6 % on day 6 and 2 ± 2 % on day 12 of differentiation (Fig. 3a, Supplementary Figure S1c). In addition, we analyzed the expression of the *Ki*-*67* gene prior to and six and 12 days after induction of differentiation following either 3D diff or 3D + T10 protocols by real-time RT-PCR (Fig. 3b), which confirmed our flow cytometry data. The effects of taxol on Ki-67-positive cells were confirmed morphologically by immunocytochemistry, where whole aggregates were fixed at different stages of differentiation and stained with antibodies against Ki-67 and the postmitotic neuronal marker, NeuN (Fig. 3c). Fewer Ki-67-positive cells and higher number of NeuN-positive cells were found in 3D + T10 samples on days 6 and 12 of differentiation in comparison with 3D diff samples. Supplementation of LUHMES differentiation medium with taxol for 48 h selectively blocked proliferation without any negative effects on neuronal cells, increased the homogeneity of the cell population, and did not interfere with further toxicological studies since taxol was washed from the cultures before toxicant exposures. Thus, we favored the 3D + T10 protocol over other differentiation conditions, and this protocol was followed as a standard differentiation protocol for further experiments.

### Characterization of LUHMES differentiation in 3D

**Fig. 4:**
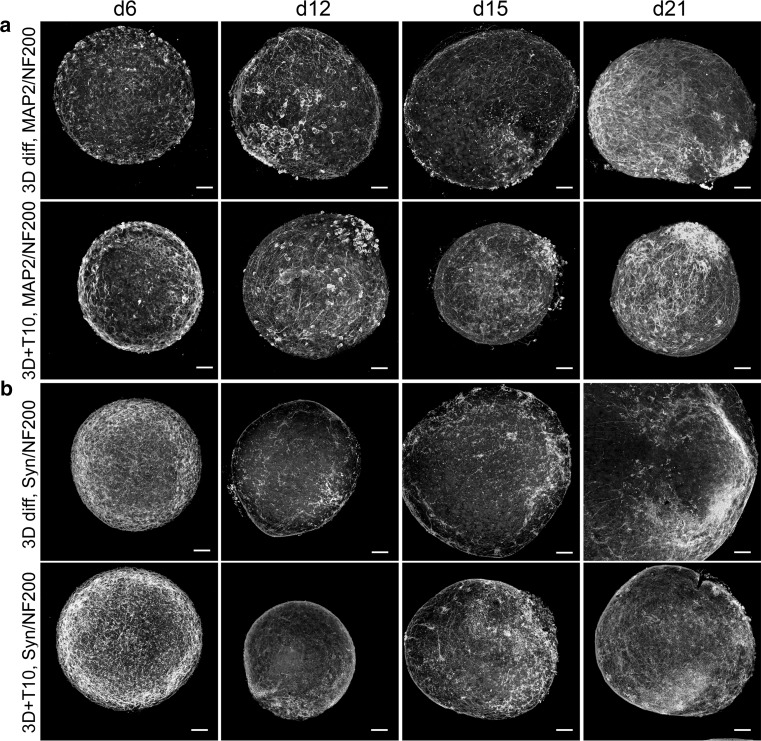
Immunocytochemistry of neuronal differentiated LUHMES in 3D diff and 3D + T10 cultures on days 6, 12, 15, and 21 after induction of differentiation. Panel **a** shows the induction of expression of MAP2 (*green*) and NF200 (*red*), as well as maturation and neurite elongation in 3D cultures followed 3D +T10 protocol versus 3D diff protocol over a span of 21 days of differentiation. Panel **b** shows the overlay of NF200 (*red*) and typical punctual staining with synaptic marker, synaptophysin (Syn, *green*). Note higher synaptophysin expression in 3D + T10 cultures versus 3D diff cultures. The nuclei were visualized with Hoechst 33342 staining. *Scale bars* are 50 μm. The aggregates were fixed on glass slides and covered with coverslips for confocal imaging which explains the larger size of the aggregates in comparison with Fig. 1, where floating aggregates were imaged (color figure online)

**Fig. 5:**
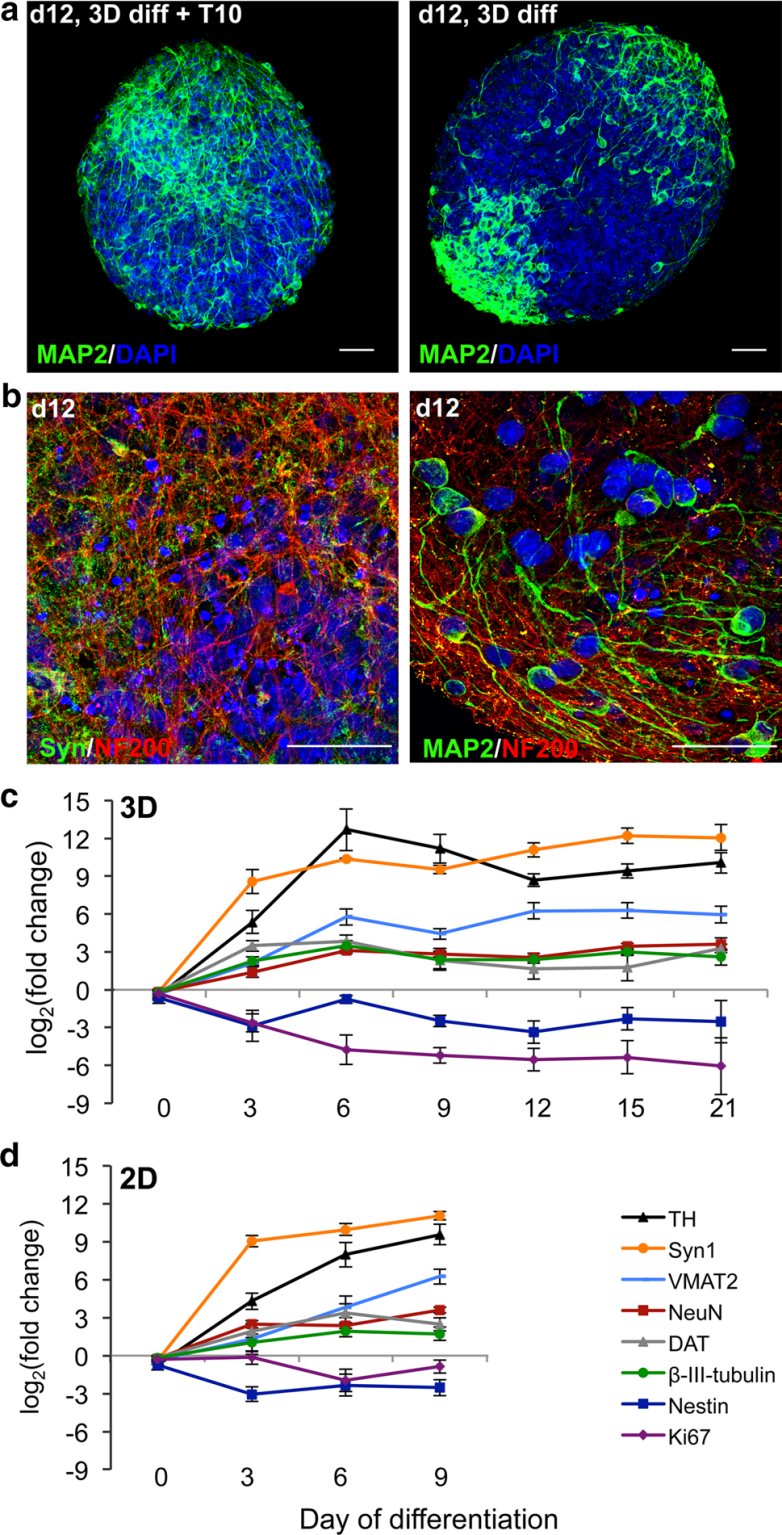
Enhanced neuronal maturation in 3D + T10 cultures. **a** MAP2 staining of representative aggregates differentiated for 12 days following either 3D +T10 (*first panel*) or 3D diff. (*second panel*) protocols. The nuclei were visualized with Hoechst 33342 staining. **b** Higher magnification (63×) of representative aggregates differentiated for 12 days under 3D + T10 conditions and stained with synaptophysin (*green*), NF200 (*red*) in the first slide and MAP2 (*green*), NF200 (*red*) in the second slide. The nuclei were visualized with Hoechst 33342 staining. *Scale bars* are 50 μm. Real-Time RT-PCR of genes involved in LUHMES neuronal differentiation and maturation. LUHMES were differentiated in 3D + T10 (**c**) and 2D monolayer cultures (**d**). RNA samples were collected on days 3, 6, 9, 12, 15, and 21 of differentiation and prior induction of differentiation (day 0) as a control. *Data* represent mean of log_2_ (fold change) ± SEM normalized to d0 from at least four independent experiments. Statistical significance was calculated using one-way ANOVA test followed by Dunnett’s post hoc test. Expression of all genes was significantly (*p* < 0.05) different in comparison with day 0, except Nestin in 3D cultures and Ki-67 in 2D cultures (Supplementary Table S2) (color figure online)

The differentiation in 3D was characterized by immunocytochemistry. In addition to Ki-67 and NeuN stainings (described above), aggregates were stained with further neuronal markers (MAP2 for dendrites and apical part of axons, neurofilament (NF200) for axons, and synaptophysin for synapses) at different stages of differentiation with (3D + T10) and without (3D diff) taxol treatment. Induction of differentiation in 3D induced the expression of MAP2, NeuN, and synaptophysin; reduced the expression of Ki-67; and changed the morphology of neurofilament- and MAP2-positive neurites (Figs. 3c, 4). Interestingly, treatment with taxol not only inhibited proliferation, but significantly enhanced maturation, dendritic morphogenesis, and arborization as shown for MAP2 and synaptophysin stainings (Figs. 4, 5a). For more detailed visualization of long neurites protruding from the differentiated neurons, high magnification of MAP2/NF200 and synaptophysin/NF200 stainings of taxol-treated aggregates is shown (Fig. 5b). These findings are in agreement with publications showing that low taxol concentrations promote lamellipodial protrusions, stabilize microtubules, and increase spine formation (Buck and Zheng 2002; Gu et al. 2008).

Real-time PCR was performed to analyze induction of neuronal genes during differentiation of LUHMES in 3D. Expression of general neuronal markers (*β*-*III*-*tubulin*, *NeuN*, *synapsin1*), marker genes specific for dopaminergic neurons [*tyrosine hydroxylase* (*TH*), *dopamine transporter* (*DAT*), and *vesicular monoamine transporter member 2* (*VMAT2*)], as well as proliferation and neural precursor markers *Ki*-*67* and *Nestin,* were analyzed in course of 3D + T10 differentiation and normalized to the expression levels at day 0 (Fig. 5c). *Ki*-*67* and *Nestin* were down-regulated during differentiation, while expression of neuronal markers was significantly induced (one-way ANOVA test followed by Dunnett’s post hoc test). The expression levels of these marker genes were similar to those in 2D differentiated LUHMES (Fig. 5d) with slightly higher expression of *TH* in 3D cultures versus 2D. Note that the expression level of all genes plateaued on day 6 of differentiation, suggesting complete differentiation.

### LUHMES 3D model for neurotoxicity testing

**Fig. 6:**
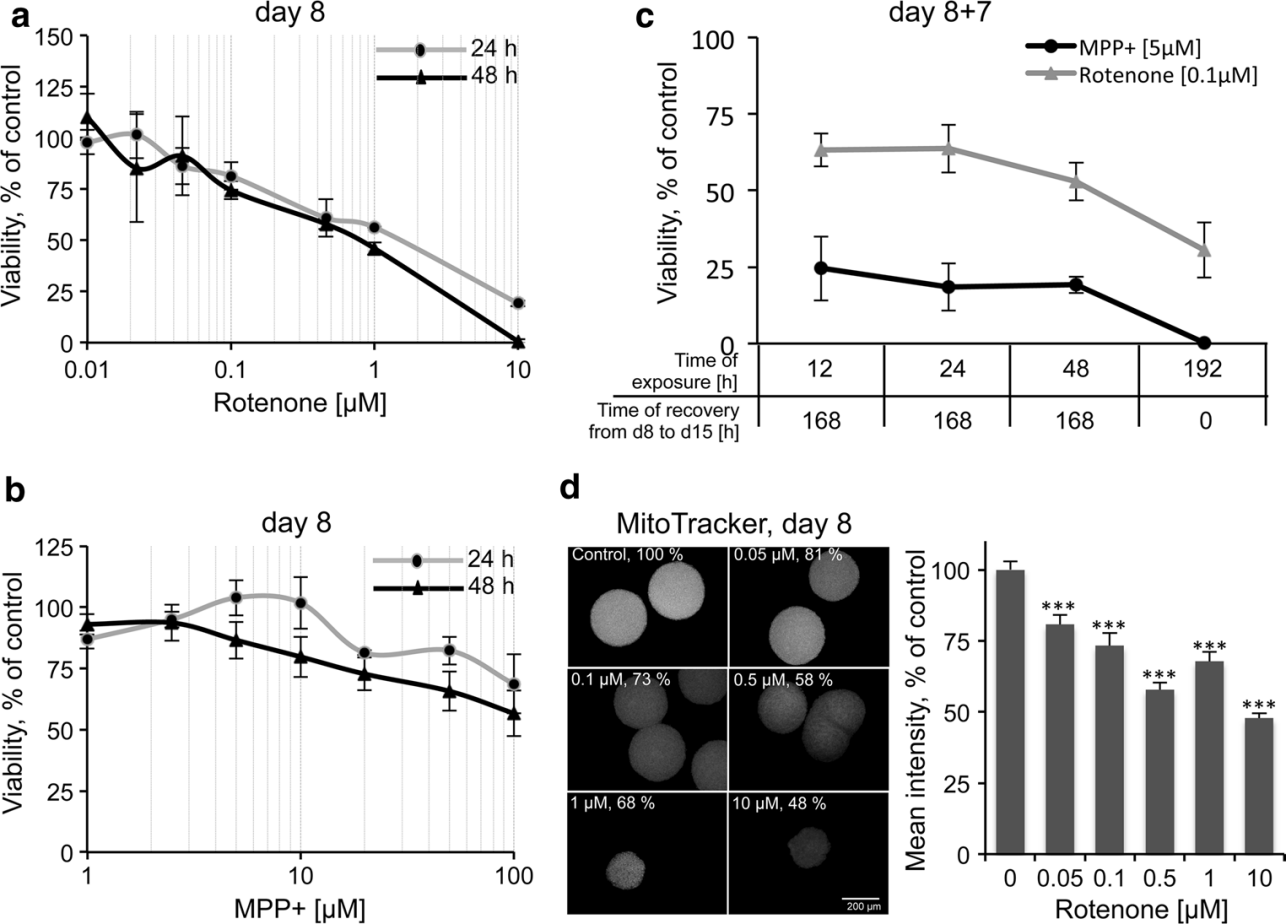
Cell viability of LUHMES aggregates after exposure to rotenone and MPP^+^. LUHMES cells were differentiated following 3D + T10 protocol and exposed reversely to different rotenone (**a**) and MPP^+^ (**b**) concentrations from day 6 to 8 (48 h) and from day 7 to 8 (24 h). **c** Cell viability after toxicant washout and recovery period. LUHMES were exposed reversely until day 8 for indicated period of time to 0.1 μM rotenone and 5 μM MPP^+^. On day 8, compounds were washed out and cells recovered for further 7 days. Chronic**/**repeat-dose (fresh substance was added with each medium exchange) exposure (192 h, from day 7 until day 15) was included as positive control. Cell viability was analyzed using resazurin reduction assay. Cell viability is presented in  % of solvent-treated controls in at least three independent experiments (*n* ≥ 3, mean ± SEM, *n* = 2 for MPP^+ ^on day 15). **d** Mitochondrial membrane potential in individual LUHMES aggregates, exposed to rotenone for 48 h from day 6 to 8 measured by Mitotracker assay. Fluorescence intensity was measured as mean *gray* values using ImageJ software and normalized to the size and then to the fluorescence intensity of DMSO control aggregates (*n* = 3), at least 10 aggregates were assayed for each independent experiment, ***p < 0.001, Kruskal–Wallis followed by Dunn’s post hoc test)

Next, we analyzed the performance of the 3D model for neurotoxicity testing by applying two well-known neurotoxicants, MPP^+^ and rotenone. Both chemicals are mitochondrial complex I inhibitors and cause Parkinsonism (Betarbet et al. 2000; Franco-Iborra et al. 2015). MPP^+^ is specific for dopaminergic neurons, because of its selective uptake by them (Langston et al. 1984), while rotenone has broader toxicity. LUHMES neuronal aggregates were treated with increasing concentrations of both compounds for 24 and 48 h. First, cell viability assay, based on mitochondria metabolic capacity, was performed to generate concentration–response curves (Fig. 6a, b). Second, a cytotoxicity assay, based on the measurement of membrane integrity, was conducted in the same samples using LDH release assay (Supplementary Figure S2). As expected, mitochondria impairment was measured at concentrations at which the cellular membrane was still intact (low LDH activity in the media), confirming the mitochondria selectivity of the test compounds by the higher sensitivity of the resazurin reduction-based assay. The concentrations (5 μM MPP^+^ and 0.1 μM rotenone) with slight mitochondria impairment after 24- and 48-h exposure were used for further gene expression and washout experiments.

As a proof of concept, compound washout experiments were performed to address counter-regulation responses after short-term exposure in comparison with long-term chronic exposure. LUHMES were differentiated in 3D and exposed to 0.1 μM rotenone and 5 μM MPP^+^ for 12, 24, 48, or 192 h. On day 8 of differentiation, after 12, 24, and 48 h of exposure, compounds were washed out, and aggregates seeded into new plates and cultivated further until day 15. In case of 192-h exposure, aggregates were exposed to the toxicants continuously from day 7 until day 15. Cell viability (resazurin reduction assay) was assessed for all exposure conditions on day 15 of differentiation (Fig. 6c). Continuous exposure (192 h) to 5 μM MPP^+^ was 100 % toxic for LUHMES, while exposure to 0.1 μM rotenone for 192 h reduced cell viability by 70 %. Interestingly, after MPP^+^ wash out, around 80 % of cells were lost by day 15. This suggested either that MPP^+^ accumulates in the aggregates and continues to affect mitochondria after wash out, or that processes initiated by 5 μM MPP^+^ cannot be reversed, and cells cannot recover from the primary hit (at least at this concentration) (Fig. 6c dark gray line).

The washout effect was different for varying durations of 0.1 μM rotenone exposures (Fig. 6c light gray line). Exposure for 12 and 24 h further reduced viability by 34 % in total, while cells treated with 0.1 μM rotenone for 48 h were more strongly affected (47 % decrease in viability). However, although 5 μM MPP^+^ was less toxic than rotenone immediately after the hit (day 8), its withdrawal could not rescue cells from ongoing cell death, while in samples treated with 0.1 μM rotenone, cell viability continued to decline but to lesser extent than in MPP^+^ samples.

Since mitochondria are the primary target for rotenone, we further evaluated the effects of rotenone on mitochondrial membrane potential in individual aggregates. LUHMES were differentiated following 3D + T10 protocol, exposed to 0.05, 0.1, and 0.5, 1 and 10 μM rotenone or DMSO from d6 to d8 of differentiation. After 48-h exposure to rotenone, the aggregates were stained with the MitoTracker dye, to allow its accumulation in mitochondria according to the magnitude of their membrane potential. The mean fluorescence intensity values were then estimated in individual aggregates by fluorescence microscopy and normalized to DMSO controls (Fig. 6d, *n* ≥ 3, independent experiments with 10–20 aggregates assayed per experiment). Mitochondrial activity was significantly reduced in rotenone-treated samples. High correlation between data from resazurin and MitoTracker assays was observed for lower rotenone concentrations (0.05, 0.1, and 0.5 μM), which was not as closely related for the higher cytotoxic concentrations (1 and 10 μM), where changes in morphology and size of the aggregates prohibited precise microscopic evaluation of MitoTracker samples.

**Fig. 7:**
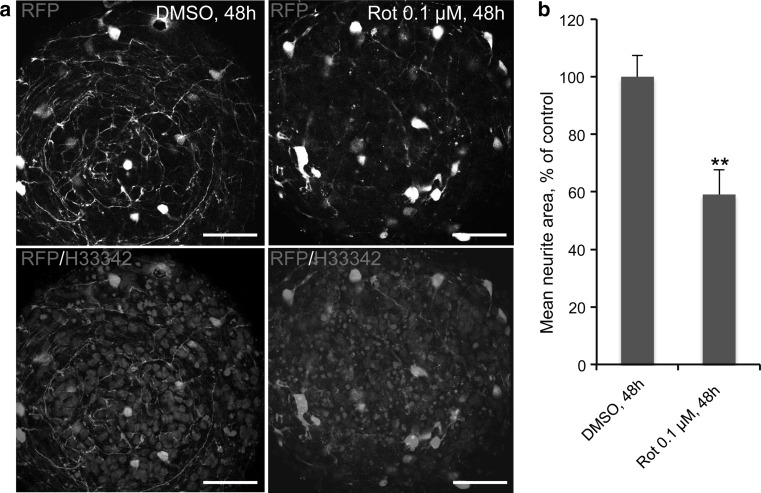
Neurite integrity in individual LUHMES aggregates exposed to rotenone. **a** Exposure to 0.1 μM rotenone for 48 h from d6 to d8 perturbed neurite integrity. Confocal images showing RFP-expressing cells (*red*) mixed in a 1:49 ratio with wild-type cells and differentiated following the 3D + T10 protocol for 8 days. Note rotenone-altered neurite integrity of viable RFP-expressing cells in comparison with DMSO control samples. Nuclei are stained with Hoechst 33342. *Scale bars* are 50 μm. **b** Quantification of neurite area in rotenone-treated samples versus DMSO controls, normalized to the number of RFP-positive cell bodies in three independent experiments (nine aggregates were quantified for rotenone-treated samples and 12 for DMSO control samples) (*n* = 3, **p < 0.01, Mann–Whitney test) (color figure online)

There are some limitations in imaging 3D cultures. Since 3D aggregates are ≥200 μm thick, imaging them using conventional fluorescence microscopy is very challenging due to issues with light scattering and penetration depth. Advanced confocal microscopy and/or two-photon microscopy in combination with optical clearing by treatment of the tissue with Sca*l*e clearing solution (Hama et al. 2011) prior to imaging overcome these limitations. Previously, it has been shown that rotenone perturbs neurite integrity in 2D LUHMES cultures (Schildknecht et al. 2013; Krug et al. 2013). To confirm these findings and to optimize the imaging of neurite integrity in 3D cultures, RFP-expressing LUHMES were used. Wild-type LUHMES were mixed with RFP-expressing LUHMES in the ratio 49:1 on day 0 of differentiation and differentiated following the 3D + T10 protocol. It was shown previously that RFP is only expressed in viable cells (Schildknecht et al. 2013). After rotenone treatment from days 6 to 8 of differentiation, RFP-expressing viable cells within the aggregates were imaged using confocal microscopy for neurite quantification. Exposure of LUHMES aggregates to 0.1 μM rotenone significantly affected the neurite integrity in comparison with DMSO controls (Fig. 7a, b). Thus, application of the fluorescent cell line mixed with wild-type cells helped to overcome the limitation of image quantification in these highly compact three-dimensional cultures.

### Exposure of the 3D LUHMES model to rotenone and MPP^+^ alters the expression of genes involved in transsulfuration and one-carbon metabolic pathways

**Fig. 8:**
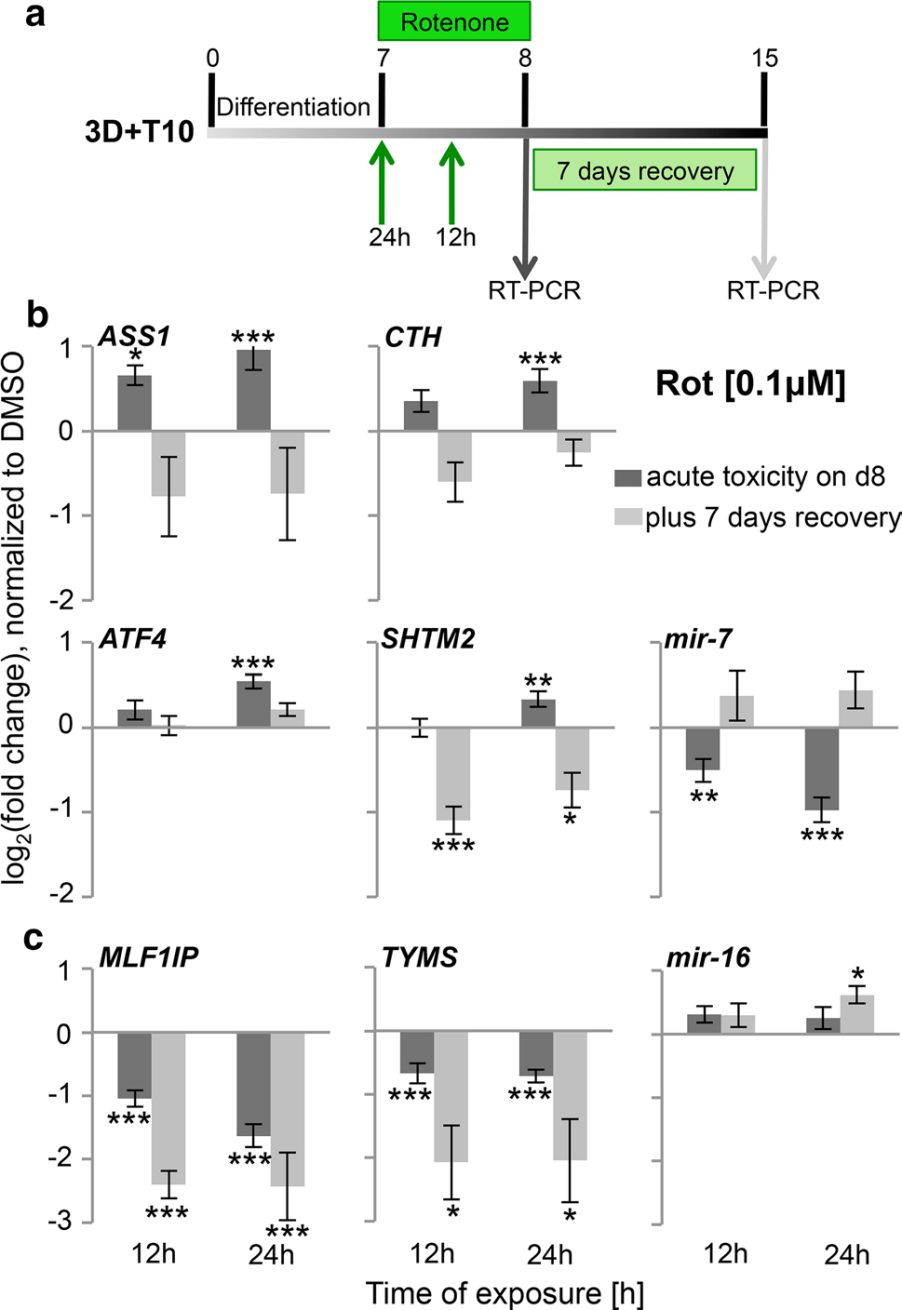
Time-dependent perturbations of gene expression after exposure of 3D LUHMES to rotenone. **a** Toxicant treatment and washout scheme: LUHMES were differentiated following 3D + T10 protocol; exposure to 0.1 μM rotenone occurred for 12 or 24 h from day 7 until day 8; Samples were collected for RT-PCR immediately after exposure on day 8 (*dark arrow*) or after rotenone washout and 7 days recovery on day 15 (*light arrow*). **b** Protein coding genes (*ASS1*, *AT4*, *CTH*, *SHMT2*) and miRNA (*mir*-*7*) with counter-regulation pattern after rotenone washout in comparison with acute response. **c** Protein coding genes (*MLF1IP*, *TYMS*) and miRNA (*mir*-*16*) with stronger response after rotenone withdrawal in comparison with acute toxicity. Dark bars show expression of the genes on day 8, while light *bars* show expression of the genes after rotenone washout and 7-day recovery. The data are means of log_2_ (fold change) ± SEM of at least three independent experiments (9–12 technical replicates). (*n* ≥ 3, *p < 0.05, **p < 0.01, and ***p < 0.001, one-way-ANOVA followed by Dunnett’s post hoc test)

The performance of the 3D model for toxicological studies was analyzed by gene expression. We have chosen a panel of candidate genes which were shown to be involved in cellular adaptation to MPP^+^ exposure in 2D LUHMES cultures by regulating central carbon metabolism and amino acid turnover (*ASS1*, argininosuccinate synthase, *SHMT2*, serine hydroxymethyl transferase), transsulfuration pathway [*CTH*, cystathionase (cystathionine γ-lyase)], oxidative stress and DNA replication and repair [*TYMS*, thymidylate synthetase *MLF1IP*, centromere protein U (MLF interacting protein)] in earlier studies (Krug et al. 2014). LUHMES were differentiated following the 3D + T10 protocol and exposed to 0.1 μM rotenone and 5 μM MPP^+^ for 12 and 24 h on day 7 (Fig. 8a). In agreement with our earlier studies (Krug et al. 2014), on day 8—immediately after exposure—we observed the same regulation trends of those genes by rotenone and MPP^+^ [Fig. 8b, c, dark bars (rotenone), Supplementary Figure S3 (MPP^+^)]. Gene expression analysis was performed in three to four independent experiments (up to 12 technical replicates) and normalized to DMSO-treated controls. *ASS1* was the most strongly up-regulated gene by MPP^+^ (FC = 3.3, 24 h) and rotenone (FC = 2.4, 24 h). *ATF4*, activating transcription factor four, was identified as upstream regulator of the cellular cascades initiated by MPP^+^ (Krug et al. 2014) but was less up-regulated in our 3D model by rotenone (FC = 1.5, 24 h), though 2.3 times increased by 24-h MPP^+^ treatment. *CTH* and *SHMT2* were more up-regulated by MPP^+^ than by rotenone. *MLF1IP* and *TYMS* were significantly down-regulated in the 3D system following MPP^+^ and rotenone treatment. As proof-of-concept experiments—to study cellular counter-regulation—rotenone was washed out on day 8 of differentiation, and cells were kept in culture for further 7 days. Since washout of 5 μM MPP^+^ did not prevent cell death, we analyzed the expression of the same panel of genes on day 15 only in rotenone-treated samples (Fig. 8b, c, light bars). Interestingly, *ASS1*, *CTH,* and *SHTM2*, which were up-regulated immediately after exposure, were down-regulated 7 days later after rotenone withdrawal (Fig. 8b), while down-regulated genes (*MLF1IP* and *TYMS*) were further repressed with an even stronger effect (Fig. 8c). *ATF4* was only slightly up-regulated on day 8 and returned to control level 7 days after recovery (Fig. 8b). This observation suggests that certain genes and signaling pathways are counter-regulated and/or may be responsible for cellular recovery after the primary hit, while other processes cannot be restored and, thus, might be a part of the new cellular homeostasis.

### Altered expression of mir-7 miRNA after exposure of 3D LUHMES to rotenone

Finally, miRNAs involved in mitochondrial functions and relevant for PD were analyzed after exposure of LUHMES aggregates to 0.1 μM rotenone. In order to test whether miRNAs are involved in the recovery process, miRNA expression was assessed on day 8 as the reaction to the primary toxicant hit and on day 15, 7 days after rotenone withdrawal (refer to Fig. 8a for treatment and sampling scheme). In agreement with the literature showing down-regulation of *mir*-*7* in PD models (Junn et al. 2009; Fragkouli and Doxakis 2014), we observed a reduction of *miR*-*7* expression as early as 12 h after rotenone treatment (Fig. 8b, dark bar), while known pro-apoptotic *miR*-*16* remained at control level (Fig. 8c). No changes were observed in expression of *miR*-*210* (hypoxia-sensitive miRNA, involved in mitochondrial respiration (Chan et al. 2012, data not shown), suggesting *mir*-*7* as a primary rotenone miRNA target prior to mitochondria-mediated apoptosis. On day 15 after rotenone washout, however, *mir*-*7* expression went back to control levels, grouping this miRNA together with other counter-regulated genes (Fig. 8b), suggesting a possible role of this miRNA in cellular adaptation and recovery. In addition, brain-specific miRNA, *mir*-*124,* was unchanged on day 8 of differentiation and was upregulated on day 15 after washout (data not shown).

## Discussion

The information gained from acute cytotoxicity studies appears to be of limited relevance for the understanding of chronic/slow-developing processes and low-dose chemical exposures (Hengstler et al. 2012). This situation also holds true for the study of neurodegenerative diseases such as Parkinson’s disease. Moreover, there are indications that the use of rodent models and cells may have low predictivity for human disease states (Hartung 2008; Leist and Hartung 2013; Leist et al. 2014), and therefore, attempts are ongoing to provide toxicological/disease models on the basis of human cells (Krause et al. 2013).

At present, there are only few options to work with human dopaminergic neurons in long-term experiments. Therefore, the primary aim of this work was to adapt the LUHMES neuronal model (Scholz et al. 2011) to 3D. Besides emerging evidence in the literature pointing out the general advantages of 3D cell systems over classical monolayer cultures (Alépée et al. 2014), the main reason for attempting the 3D LUHMES model here was the opportunity for prolonged cultivation and increased cell survival after induction of differentiation. The 2D LUHMES model has been used successfully to study neurotoxicity, especially related to Parkinson’s (Schildknecht et al. 2009; Stiegler et al. 2011; Schildknecht et al. 2013; Krug et al. 2013, 2014; Zhang et al. 2014; Stępkowski et al. 2015). Several neurotoxicological end points were established for monolayer LUHMES cultures, such as neurite outgrowth (Krug et al. 2013), Tau phosphorylation and associated cell death (Selenica et al. 2007), reporter cell line-based assays using high-content imaging (Schildknecht et al. 2013; van Vliet et al. 2014), omics technologies to study perturbations in cellular metabolism, and gene expression after toxicant exposure (Krug et al. 2014). However, in prolonged three-dimensional cultures, cellular junctions are more in vivo-like conditions and allow pronounced neuronal network formation. In addition, the specific composition of the extracellular matrix in the brain, which differs significantly from that of other organs (Yamaguchi 2000) and was shown to promote synaptogenesis and neuronal network formation (Dityatev and Fellin 2008; Frischknecht and Gundelfinger 2012), and the lack of glia support, may make 2D neuronal cultures more sensitive and less adherent to cell culture plastic ware.

We adapted the LUHMES differentiation protocol to 3D applying a gyratory shaking technic (Honegger and Monnet-Tschudi 2001) and showed prolonged survival in 3D in comparison with 2D cultures (Fig. 1b, c). A frequent concern about 3D cultures is the potential insufficient nutrient and oxygen supply to the center of aggregates (Minchinton and Tannock 2006; Derda et al. 2009). We demonstrated that the gyratory shaking method of LUHMES spheroid cultivation combined with taxol treatment allows controlled spheroid size and fast penetration of compounds, for the example of Hoechst 33342 dye, into the middle of aggregates. Nuclei staining with Hoechst 33342, as well as caspase 3/7 staining, ensured the absence of cell death in the center of aggregates during differentiation (Fig. 2a–c). The absence of proliferating cells after 48-h taxol treatment (Fig. 3) also proved the efficient penetration of the drug throughout the aggregates. Demonstration of penetration of relatively large molecules such as positive-charged Hoechst 33342 (Mw = 616 g/mol) and uncharged taxol (Mw = 854 g/mol) throughout the aggregates ensures that the model is suitable for compound testing.

LUHMES cells formed a pronounced neuronal network and continued to mature further in 3D at later days of differentiation as shown in Fig. 4a, b (note increase in synaptophysin-stained cells, as well as advanced neuronal network from day 12 to day 21 of differentiation), showing the importance of keeping cultures longer for neuronal network formation and synaptogenesis studies. Although the LUHMES 3D model consists of homotypic aggregates, we were surprised to observe cellular organization and polarity within the aggregates similar to organotypical spheroids described by (Lancaster et al. 2013) as shown in Figs. 4a, 5a for MAP2-positive cells, accumulating at one side of the aggregate starting on day 12 and onwards. This observation suggests that MAP2-positive cells may migrate from the initial differentiation area through the aggregate during maturation. A similar pattern was also observed for synaptophysin staining but not for NF200, which was equally distributed throughout the aggregates at all stages of differentiation (Fig. 4a, b). Further experiments using combinations of live imaging techniques and RFP/GFP expression LUHMES may clarify this observation.

Especially for long-term exposures in vivo (where organ toxicities are recorded), the first organ to decompensate leads the toxicity. Therefore, in vitro it is important to measure not only the reaction of cells to a hard, single hit, if we want to evaluate neurodegenerative processes or degenerative diseases. Instead, we should rather measure the potential to compensate/recover from multiple subtoxic hits, and over longer periods. Such differences are important to work out to follow the new toxicological strategies suggested by the national research council (US) in 2007 (NRC 2007). Thus, the long-term shelf life of our model may allow designing such experiments.

In this study, we used the well-known dopaminergic neuronal toxicants MPP^+^ and rotenone as model compounds. MPP^+^ is a toxic metabolite of 1-methyl-4-phenyl-1.2.3.6-tetrahydropyridine (MPTP), and rotenone is a broadly used pesticide. Both MPTP and rotenone are highly lipophilic, which makes it very easy for them to cross the blood–brain barrier, and they represent prime examples of mitochondrial toxins (Miller et al. 2009). Both compounds accumulate in mitochondria and inhibit complex I of the electron transport (respiratory) chain, a major target of ROS and the reason they are used in animal models to study Parkinsonism (Langston et al. 1984; Betarbet et al. 2000). As a proof of concept, LUHMES were differentiated in 3D, exposed either to MPP^+^ or rotenone for a short period of time (12, 24, or 48 h); then, the compounds were washed out, and cells were cultivated for a further 7 days. First, we observed a similar cytotoxicity on day 8 in 3D as in our earlier studies in monolayer cultures (Fig. 6a, b; Krug et al. 2014), as measured by the resazurin reduction assay. Second, we confirmed perturbation of genes involved in one-carbon metabolism and transsulfuration pathways (*ASS1*, *CTH,* and *SHTM2*), on day 8 after 12 or 24 h of exposure to MPP^+^ and rotenone (Fig. 8b, c; Supplementary Figure S3; Krug et al. 2014). Interestingly, MPP^+^ effects were stronger on certain genes than those of rotenone (much stronger induction of *ASS1* and *AT4*, *SHMT2,* and *CTH*, for example). Third, we showed a down-regulation of PD-relevant *mir*-*7* as early as 12 h after rotenone exposure, while pro-apoptotic *mir*-*16* and rotenone-sensitive *mir*-*210* were not yet significantly perturbed (Fig. 8b, c and data not shown). Finally, washout experiments demonstrated different counter-regulation after short-term exposures to sub-cytotoxic concentrations of rotenone or MPP^+^. The 3D cultures allow moving the aggregates into a new cell culture dish, thus avoiding remaining toxicant contamination (e.g., compound bout to plastic), which represents an enormous advantage over 2D cultures for resilience studies. Interestingly, 5 μM MPP^+^ was not cytotoxic 24 h after exposure, but MPP^+^ withdrawal was not sufficient, and 80 % of cells were dead after the 7-day recovery period, likely due to the accumulation of MPP^+^ within the cells. 0.1 μM rotenone decreased cell viability by 20 % after 24 h, which was further decreased by additional 16 % after rotenone withdrawal (Fig. 6c). The molecular mechanisms, which prevented 64 % of cells from death in rotenone samples after washout, shall be addressed in future experiments. We observed different patterns of gene expression after 7 days of recovery. Genes down-regulated by rotenone, *MLF1IP* and *TYMS,* were further down-regulated, while *mir*-*7* returned to normal level with a slight tendency for up-regulation. *mir*-*7* is one of the few known miRNAs related to PD (reviewed in Mouradian 2012; Kaidery et al. 2013). One of *mir*-*7*’s confirmed targets is *α*-synuclein, a major player in PD pathogenesis (Junn et al. 2009). Overexpression of *mir*-*7* in murine primary cortical neurons prior to exposure to MPP^+^ demonstrated a neuroprotective effect against MPP^+^ through activation of TOR pathway (Fragkouli and Doxakis 2014). Recently, *mir*-*7* was shown to protect SH-SY5Y cells against MPP^+^-induced cell death by targeting RelA (a component of nuclear factor-κB (NF-κB) (Choi et al. 2014). These studies, together with our findings, suggest a possible role of this miRNA in counter-regulation against the stress and for cellular resilience after exposure to the mitochondria toxicants MPP^+^ and rotenone. Here, for the first time, we demonstrated a significant down-regulation of *mir*-*7* expression by rotenone and its complete recovery after rotenone withdrawal in a human-relevant Parkinson’s disease in vitro model. Further functional studies are needed to confirm this observation. In contrast, rotenone-induced *ASS1*, *SHTM2,* and *CTH* were down-regulated after rotenone withdrawal (Fig. 8b), which could also be involved in counter-regulation mechanisms.

A further advantage of the longer-lived 3D LUHMES model over monolayer cultures could be the easier co-culture and readout of LUHMES with other cell types, e.g., astrocytes or liver cells. 3D aggregates can be added to monolayer cultures of astrocytes or liver cells for the time of exposure without mixing the two cell populations as with 2D cultures (Efrémova et al. 2015). After exposure, the response to the toxicant treatment can be assessed separately for LUHMES spheres and the other cell types. This may allow for the study of neuroprotective effects of factors released by glial cells and inclusion of metabolic competence (liver cells) in the model.

In addition, the 3D LUHMES model may allow us to study the protective effects of Parkinson’s drug therapies by first exposing the cells to the toxicant and then to the drug. Thus, the model may have the potential for restorative/disease-modifying drug screening. Using reporter cell lines, in combination with quantitative high-content imaging, may contribute significantly to Parkinson’s drug screening (Schildknecht et al. 2013). The introduction of the fluorescent cell lines into the wild-type LUHMES population in low percentage allows clear visualization of neurites and their assignment to the corresponding cell bodies. Quantification of cellular and neurite morphology in the mixed cultures of fluorescent cell lines, together with wild-type LUHMES as described in our proof-of-concept experiment in Fig. 7, can be used for the fast screening of potential toxicants (van Vliet et al. 2014) contributing to disease development as well as the efficiency of newly developed treatments.

In conclusion, we have established a 3D LUHMES model that will allow analysis of the long-term effects of toxicant exposure, such as delayed response to the toxicant insult, cellular resilience, and/or adaptation to a new homeostasis after toxicant withdrawal (discussed in Smirnova et al. 2015b).

## Electronic supplementary material

Below is the link to the electronic supplementary material.

Supplementary material 1 (PDF 8102 kb)

Supplementary material 2 (DOCX 17 kb)

## References

[CR1] Alépée N, Bahinski A, Daneshian M (2014). State-of-the-art of 3D cultures (organs-on-a-chip) in safety testing and pathophysiology. ALTEX.

[CR2] Ascherio A, Chen H, Weisskopf MG, O’Reilly E, McCullough ML, Calle EE, Schwarzschild MA, Thun MJ (2006). Pesticide exposure and risk for Parkinson’s disease. Ann Neurol.

[CR3] Bandiera S, Matégot R, Girard M (2013). MitomiRs delineating the intracellular localization of microRNAs at mitochondria. Free Radic Biol Med.

[CR4] Betarbet R, Sherer TB, MacKenzie G (2000). Chronic systemic pesticide exposure reproduces features of Parkinson’s disease. Nat Neurosci.

[CR5] Borland MK, Trimmer PA, Rubinstein JD (2008). Chronic, low-dose rotenone reproduces Lewy neurites found in early stages of Parkinson’s disease, reduces mitochondrial movement and slowly kills differentiated SH-SY5Y neural cells. Mol Neurodegener.

[CR6] Buck KB, Zheng JQ (2002). Growth cone turning induced by direct local modification of microtubule dynamics. J Neurosci.

[CR7] Chan YC, Banerjee J, Choi SY, Sen CK (2012). miR-210: the master hypoxamir. Microcirculation.

[CR8] Chinta SJ, Andersen JK (2005). Dopaminergic neurons. Int J Biochem Cell Biol.

[CR9] Choi DC, Chae Y-J, Kabaria S (2014). MicroRNA-7 protects against 1-methyl-4-phenylpyridinium-induced cell death by targeting RelA. J Neurosci.

[CR10] Constantinescu R, Constantinescu AT, Reichmann H, Janetzky DB (2007). Neuronal differentiation and long-term culture of the human neuroblastoma line SH-SY5Y. Neuropsychiatric disorders an integrative approach.

[CR11] Costello S, Cockburn M, Bronstein J (2009). Parkinson’s disease and residential exposure to maneb and paraquat from agricultural applications in the central valley of California. Am J Epidemiol.

[CR12] Derda R, Laromaine A, Mammoto A (2009). Paper-supported 3D cell culture for tissue-based bioassays. Proc Natl Acad Sci USA.

[CR13] Dityatev A, Fellin T (2008). Extracellular matrix in plasticity and epileptogenesis. Neuron Glia Biol.

[CR14] Efrémova L, Schildknecht S, Adam M (2015). Prevention of the degeneration of human dopaminergic neurons in an astrocyte co-culture system allowing endogenous drug metabolism. Br J Pharmacol.

[CR15] Fragkouli A, Doxakis E (2014). miR-7 and miR-153 protect neurons against MPP(+)-induced cell death via upregulation of mTOR pathway. Front Cell Neurosci.

[CR16] Franco-Iborra S, Vila M, Perier C (2015). The Parkinson disease mitochondrial hypothesis: where are we at?. Neuroscientist.

[CR17] Frischknecht R, Gundelfinger ED (2012). The brain’s extracellular matrix and its role in synaptic plasticity. Adv Exp Med Biol.

[CR18] Fujita KA, Ostaszewski M, Matsuoka Y (2014). Integrating pathways of Parkinson’s disease in a molecular interaction map. Mol Neurobiol.

[CR19] Gerhardt E, Kügler S, Leist M (2001). Cascade of caspase activation in potassium-deprived cerebellar granule neurons: targets for treatment with peptide and protein inhibitors of apoptosis. Mol Cell Neurosci.

[CR20] Giraldez AJ, Cinalli RM, Glasner ME (2005). MicroRNAs regulate brain morphogenesis in zebrafish. Science.

[CR21] Grau CM, Greene LA (2012). Use of PC12 cells and rat superior cervical ganglion sympathetic neurons as models for neuroprotective assays relevant to Parkinson’s disease. Methods Mol Biol.

[CR22] Greene LA, Tischler AS (1976). Establishment of a noradrenergic clonal line of rat adrenal pheochromocytoma cells which respond to nerve growth factor. Proc Natl Acad Sci USA.

[CR23] Gu J, Firestein BL, Zheng JQ (2008). Microtubules in dendritic spine development. J Neurosci.

[CR24] Hama H, Kurokawa H, Kawano H (2011). Scale: a chemical approach for fluorescence imaging and reconstruction of transparent mouse brain. Nat Neurosci.

[CR90] Hartung T and Leist M (2008) Food for thought ... on the evolution of toxicology and the phasing out of animal testing. ALTEX 25(2):91-102 10.14573/altex.2008.2.91 18551232

[CR25] Hartung T (2014). 3D: a new dimension of in vitro research. Adv Drug Deliv Rev.

[CR26] Henchcliffe C, Beal MF (2008). Mitochondrial biology and oxidative stress in Parkinson disease pathogenesis. Nat Clin Pract Neurol.

[CR27] Hengstler JG, Marchan R, Leist M (2012). Highlight report: towards the replacement of in vivo repeated dose systemic toxicity testing. Arch Toxicol.

[CR91] Hogberg, HT, Bressler, J, Christian, KM, Harris, G, Makri, G, O'Driscoll, C, et al. (2013) Toward a 3D model of human brain development for studying gene/environment interactions. Stem Cell Research & Therapy 4 Suppl 1, S4–S4. doi:10.1186/scrt365 10.1186/scrt365 PMC4029162 24564953

[CR28] Honegger P, Monnet-Tschudi F (2001). Aggregating neural cell cultures. Protocols for neural cell culture.

[CR29] Hu W, He Y, Xiong Y (2015). Derivation, expansion, and motor neuron differentiation of human-induced pluripotent stem cells with non-integrating episomal vectors and a defined xenogeneic-free culture system. Mol Neurobiol.

[CR30] Huang W, Li MD (2009). Nicotine modulates expression of miR-140*, which targets the 3′-untranslated region of dynamin 1 gene (Dnm1). Int J Neuropsychopharmacol.

[CR31] Huang TT, Liu YY, Huang MM (2010). Wnt1-cre-mediated conditional loss of Dicer results in malformation of the midbrain and cerebellum and failure of neural crest and dopaminergic differentiation in mice. Fen Zi Xi Bao Sheng Wu Xue Bao.

[CR32] Junn E, Lee K-W, Jeong BS, Chan TW, Im JY, Mouradian MM (2009). Repression of alpha-synuclein expression and toxicity by microRNA-7. Proc Natl Acad Sci USA.

[CR33] Kaidery NA, Tarannum S, Thomas B (2013). Epigenetic landscape of Parkinson’s disease: emerging role in disease mechanisms and therapeutic modalities. Neurotherapeutics.

[CR34] Kim JH, Auerbach JM, Rodriguez-Gomez JA (2002). Dopamine neurons derived from embryonic stem cells function in an animal model of Parkinson’s disease. Nature.

[CR35] Kim J, Inoue K, Ishii J (2007). A MicroRNA feedback circuit in midbrain dopamine neurons. Science.

[CR36] Kim JH, Park SG, Song S-Y (2013). Reactive oxygen species-responsive miR-210 regulates proliferation and migration of adipose-derived stem cells via PTPN2. Cell Death Dis.

[CR37] Krause K-H, van Thriel C, De Sousa PA (2013). Monocrotophos in Gandaman village: India school lunch deaths and need for improved toxicity testing. Arch Toxicol.

[CR38] Krug AK, Balmer NV, Matt F (2013). Evaluation of a human neurite growth assay as specific screen for developmental neurotoxicants. Arch Toxicol.

[CR39] Krug AK, Gutbier S, Zhao L (2014). Transcriptional and metabolic adaptation of human neurons to the mitochondrial toxicant MPP(+). Cell Death Dis.

[CR40] Kumar Singh N, Dev Banerjee B, Bala K (2014). Gene–gene and gene-environment interaction on the risk of Parkinson disease. Curr Aging Sci.

[CR41] Lancaster MA, Renner M, Martin C-A (2013). Cerebral organoids model human brain development and microcephaly. Nature.

[CR42] Langston JW, Langston EB, Irwin I (1984). MPTP-induced parkinsonism in human and non-human primates–clinical and experimental aspects. Acta Neurol Scand Suppl.

[CR43] Lau P, de Strooper B (2010). Dysregulated microRNAs in neurodegenerative disorders. Semin Cell Dev Biol.

[CR44] Lee J-W, Cannon JR (2015). LRRK2 mutations and neurotoxicant susceptibility. Exp Biol Med (Maywood).

[CR92] Leist M, Hartung T (2013) Inflammatory findings on species extrapolations: humans are definitely no 70-kg mice. Arch Toxicol 87(4):563-7 doi:10.1007/s00204-013-1038-0 10.1007/s00204-013-1038-0 PMC3604596 23503654

[CR93] Leist M, Hasiwa N, Rovida C, Daneshian M, Basketter D, Kimber I, Clewell H, Gocht T, Goldberg A, Busquet F, Rossi AM, Schwarz M, Stephens M, Taalman R, Knudsen TB, McKim J, Harris G, Pamies D, Hartung T (2014) Consensus report on the future of animal-free systemic toxicity testing. ALTEX 31(3):341-56. doi:10.14573/altex.1406091 10.14573/altex.1406091 25061899

[CR45] Leucht C, Stigloher C, Wizenmann A (2008). MicroRNA-9 directs late organizer activity of the midbrain-hindbrain boundary. Nat Neurosci.

[CR46] Li X, Jin P (2010). Roles of small regulatory RNAs in determining neuronal identity. Nat Publ Group.

[CR47] Li P, Jiao J, Gao G, Prabhakar BS (2012). Control of mitochondrial activity by miRNAs. J Cell Biochem.

[CR48] Lingor P, Unsicker K, Krieglstein K (1999). Midbrain dopaminergic neurons are protected from radical induced damage by GDF-5 application. J Neural Transm.

[CR49] Lotharius J, Falsig J, van Beek J (2005). Progressive degeneration of human mesencephalic neuron-derived cells triggered by dopamine-dependent oxidative stress is dependent on the mixed-lineage kinase pathway. J Neurosci.

[CR50] Maertens A, Luechtefeld T, Kleensang A, Hartung T (2015). MPTP’s pathway of toxicity indicates central role of transcription factor SP1. Arch Toxicol.

[CR51] Miller RL, Miller RL, James-Kracke M (2009). Oxidative and inflammatory pathways in Parkinson’s disease. Neurochem Res.

[CR52] Minchinton AI, Tannock IF (2006). Drug penetration in solid tumours. Nat Rev Cancer.

[CR53] Miranda RC, Pietrzykowski AZ, Tang Y (2010). MicroRNAs: master regulators of ethanol abuse and toxicity?. Alcohol Clin Exp Res.

[CR54] Mouradian MM (2012). MicroRNAs in Parkinson’s disease. Neurobiol Dis.

[CR55] NRC—National Research Council, Committee on Toxicity Testing and Assessment of Environmental Agents (2007). Toxicity testing in the 21st century: a vision and a strategy.

[CR56] Pallocca G, Fabbri M, Sacco MG (2013). miRNA expression profiling in a human stem cell-based model as a tool for developmental neurotoxicity testing. Cell Biol Toxicol.

[CR57] Rahnenführer J, Leist M (2015). From smoking guns to footprints: mining for critical events of toxicity pathways in transcriptome data. Arch Toxicol.

[CR58] Saba R, Störchel PH, Aksoy-Aksel A (2012). Dopamine-regulated microRNA MiR-181a controls GluA2 surface expression in hippocampal neurons. Mol Cell Biol.

[CR59] Schildknecht S, Pöltl D, Nagel DM (2009). Requirement of a dopaminergic neuronal phenotype for toxicity of low concentrations of 1-methyl-4-phenylpyridinium to human cells. Toxicol Appl Pharmacol.

[CR60] Schildknecht S, Karreman C, Pöltl D (2013). Generation of genetically-modified human differentiated cells for toxicological tests and the study of neurodegenerative diseases. ALTEX.

[CR61] Schlachetzki JCM, Saliba SW, de Oliveira ACP (2012). Studying neurodegenerative diseases in culture models. Rev Bras Psiquiatr.

[CR62] Schmittgen TDT, Livak KJK (2008). Analyzing real-time PCR data by the comparative C(T) method. Nat Protoc.

[CR63] Scholz D, Pöltl D, Genewsky A (2011). Rapid, complete and large-scale generation of post-mitotic neurons from the human LUHMES cell line. J Neurochem.

[CR64] Selenica M-L, Jensen HS, Larsen AK (2007). Efficacy of small-molecule glycogen synthase kinase-3 inhibitors in the postnatal rat model of tau hyperphosphorylation. Br J Pharmacol.

[CR65] Smirnova L, Sittka A, Luch A (2012). On the role of low-dose effects and epigenetics in toxicology. EXS.

[CR66] Smirnova L, Block K, Sittka A (2014). MicroRNA profiling as tool for in vitro developmental neurotoxicity testing: the case of sodium valproate. PLoS ONE.

[CR67] Smirnova L, Seiler AEM, Luch A (2015). microRNA profiling as tool for developmental neurotoxicity testing (DNT). Curr Protoc Toxicol.

[CR68] Smirnova L, Harris G, Leist M, Hartung T (2015). Cellular resilience. ALTEX.

[CR69] Srikanth P, Young-Pearse TL (2014). Stem cells on the brain: modeling neurodevelopmental and neurodegenerative diseases using human induced pluripotent stem cells. J Neurogenet.

[CR70] Stępkowski TM, Wasyk I, Grzelak A, Kruszewski M (2015). 6-OHDA-induced changes in Parkinson’s disease-related gene expression are not affected by the overexpression of PGAM5 in in vitro differentiated embryonic mesencephalic cells. Cell Mol Neurobiol.

[CR71] Stiegler NV, Krug AK, Matt F, Leist M (2011). Assessment of chemical-induced impairment of human neurite outgrowth by multiparametric live cell imaging in high-density cultures. Toxicol Sci.

[CR72] Tal TL, Tanguay RL (2012). Non-coding RNAs: novel targets in neurotoxicity. Neurotoxicology.

[CR73] Tanner CM, Kamel F, Ross GW (2011). Rotenone, paraquat, and Parkinson’s disease. Environ Health Perspect.

[CR74] Todorovic M, Newman JRB, Shan J, Bentley S, Wood SA, Silburn PA, Mellick GD (2014). Comprehensive assessment of genetic sequence variants in the antioxidant ‘master regulator’ nrf2 in idiopathic Parkinson’s disease. PLoS ONE.

[CR75] van Vliet EE, Morath SS, Eskes CC (2008). A novel in vitro metabolomics approach for neurotoxicity testing, proof of principle for methyl mercury chloride and caffeine. Neurotoxicology.

[CR76] van Vliet E, Daneshian M, Beilmann M (2014). Current approaches and future role of high content imaging in safety sciences and drug discovery. ALTEX.

[CR77] Volbracht C, van Beek J, Zhu C (2006). Neuroprotective properties of memantine in different in vitro and in vivo models of excitotoxicity. Eur J Neurosci.

[CR78] Wang A, Costello S, Cockburn M (2011). Parkinson’s disease risk from ambient exposure to pesticides. Eur J Epidemiol.

[CR79] Wheeler HE, Wing C, Delaney SM (2015). Modeling chemotherapeutic neurotoxicity with human induced pluripotent stem cell-derived neuronal cells. PLoS ONE.

[CR80] Yamaguchi Y (2000). Lecticans: organizers of the brain extracellular matrix. Cell Mol Life Sci.

[CR81] Yang D, Li T, Wang Y (2012). miR-132 regulates the differentiation of dopamine neurons by directly targeting Nurr1 expression. J Cell Sci.

[CR82] Zhang X-M, Yin M, Zhang M-H (2014). Cell-based assays for Parkinson’s disease using differentiated human LUHMES cells. Acta Pharmacol Sin.

