# Supplementary material for: A LUHMES 3D dopaminergic neuronal model for neurotoxicity testing allowing long-term exposure and cellular resilience analysis
Source: Arch Toxicol. 2015 Dec 8;90(11):2725–43. doi: 10.1007/s00204-015-1637-z (PMC5065586; doi:10.1007/s00204-015-1637-z)
Supplement: Supplementary file 1 — Supplementary material 1 (PDF 8102 kb) [file 204_2015_1637_MOESM1_ESM.pdf]

# A LUHMES 3D dopaminergic neuronal model for neurotoxicity testing allowing long-term exposure and cellular resilience analysis

Smirnova L.[1#], Harris G.[1], Delp J.[2], Valadares M.[1], Pamies D.[1], Hogberg HT.[1], Waldmann T.[2], Leist M.[2], and Hartung T.[1,2]

[1]Center for Alternatives to Animal Testing (CAAT), Johns Hopkins University, Bloomberg School of Public Health, Baltimore, USA

[2]Center for Alternatives to Animal Testing (CAAT), Department of Biology, University of Konstanz, Konstanz, Germany

[#] Corresponding author: lsmirno1@jhu.edu, Tel: +1 (410) 614-4980, fax: +1 410 (614) 28-71

# Supplementary material

**Supplementary Table S1. SYBR Green PCR primer sequences.**

| Name | Forward sequence, 5'-3' | Reverse Sequence, 5'-3' |
|---|---|---|
| ASS1 | TGCTCCCTGGAGGATGCCTG | GTGTAGAGACCTGGAGGCGC |
| ATF4 | GGCTGGCTGTGGATGGGTTG | CTCCTGGACTAGGGGGGCAA |
| CBS | TCCTGGGAATGGTGACGCTT | GTGCTGTGGTACTGGATCTG |
| CTH | TGGATGATGTGTATGGAGGTACAAACAGG | GCCTTCAATGTCAATCACCTTCTGGG |
| GAPDH | CACCATCTTCCAGGAGCGAGATC | GCAGGAGGCATTGCTGATGATC |
| MLF1IP | TTTGTAAGGCAGCCATCGCC | CTGTGGCTCTAACCGAAGCA |
| SHMT2 | CAACCTGGCACTGACTGCTC | GATGTCCGCGTGCTTGAAAG |
| TYMS | CAGCTTCAGCGAGAACCCAG | ACCTCGGCATCCAGCCCAAC |

**Supplementary Table S 2. p values for statistical significant changes in neural gene expression upon induction of differentiation in 3D and 2D cultures**: one-way ANOVA test followed by Dunnett's post-hoc test. p-value <0.05 is denoted in the table by *, p<0.01 by **, and p<0.001 by ***, respectively

| | 3D + T10 differentiation | | | | | | 2D differentiation | | |
|---|---|---|---|---|---|---|---|---|---|
| genes \ days | 3 | 6 | 9 | 12 | 15 | 21 | 3 | 6 | 9 |
| TH | *** | *** | *** | *** | *** | *** | *** | *** | *** |
| Syn1 | *** | *** | *** | *** | *** | *** | *** | *** | *** |
| VMAT2 | n.s. | *** | *** | *** | *** | *** | n.s. | ** | *** |
| NeuN | ** | *** | *** | *** | *** | *** | *** | *** | *** |
| DAT | * | ** | * | n.s. | n.s. | * | * | *** | *** |
| β-III-tub | *** | *** | *** | *** | *** | *** | n.s. | ** | ** |
| Nestin | n.s. | n.s. | n.s. | n.s. | n.s. | n.s. | * | n.s. | * |
| Ki67 | n.s. | * | ** | ** | *** | ** | n.s. | n.s. | n.s. |

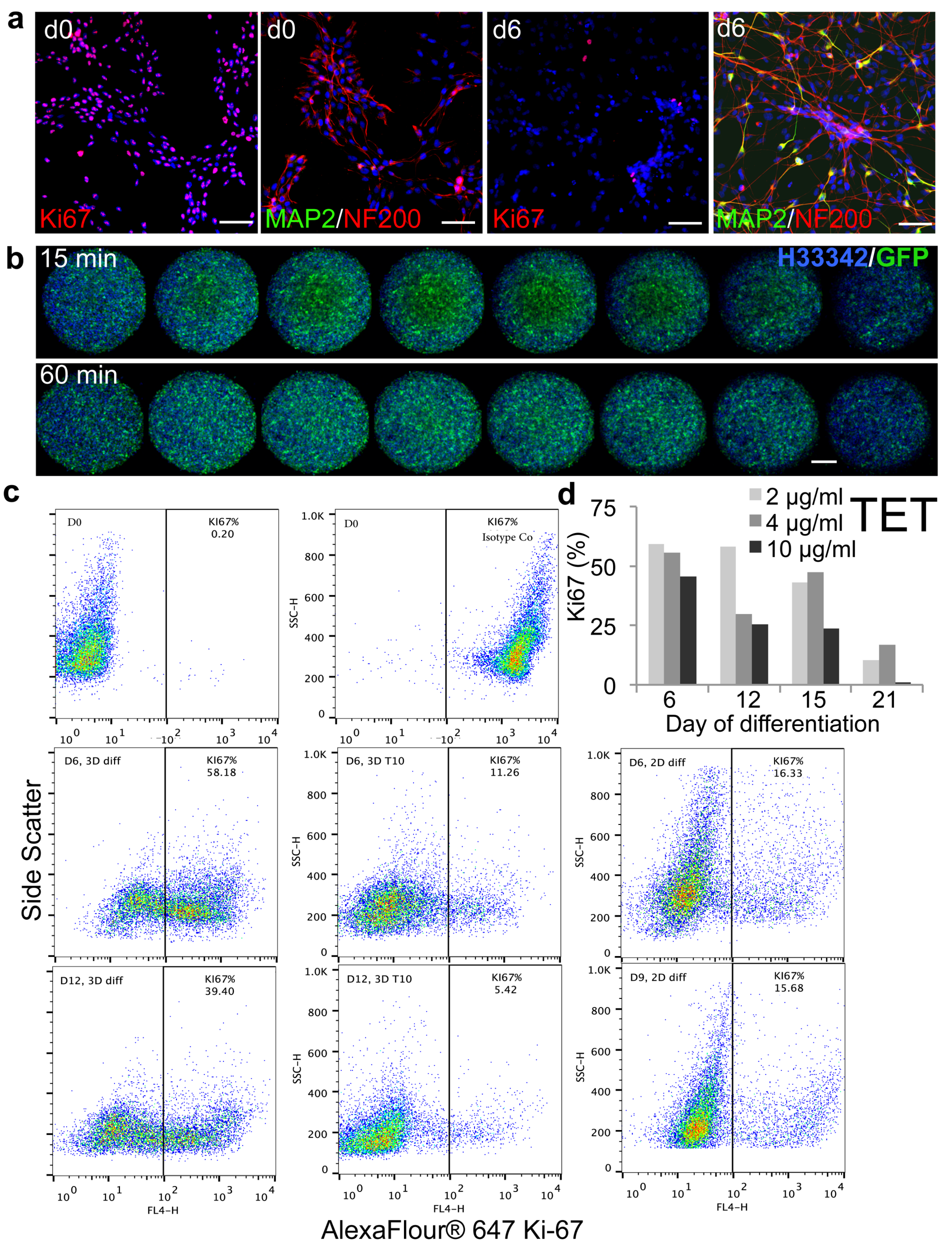


**Supplementary Figure S1** **a** Two first photographs show undifferentiated LUHMES expressing Ki-67, neurofilament (NF200), but not MAP2. Two further photographs in panel **a** show changes in morphology upon induction of differentiation in 2D, induction of MAP2 expression, and reduction of Ki-67 expression. *Scale* bar is 50 µm **b** Penetration assay with Hoechst 33342: 12-day-old aggregates of GFP-expressing LUHMES were differentiated according to 3D diff protocol and were stained with Hoechst 33342. Montages of confocal optical slices of the aggregates after 15 and 60 min incubation with Hoechst 33342 are shown to demonstrate time-dependent penetration of Hoechst 33342 through the aggregates. *Scale* bar is 100 µm. **c** Proliferation rate in course of differentiation in 2D, 3D diff and 3D+T10 treatment based on Ki-67 expression, measured by flow cytometry. Representative dot plots (Side Scatter vs. Fluorescence 4 (Alexa-647)) for both 3D conditions are shown for days 6 and 12 and for 2D cultures for days 6 and 9. Ki-67 expression in undifferentiated LUHMES (d0) was more than 99% and was used together with isotype control antibody staining to set the gates. **d** Effects of increased tetracycline concentration on Ki-67 expression in neuronal-differentiated LUHMES in 3D. LUHMES were differentiated following 3D diff protocol in differentiation medium supplemented with 2, 4 or 8 µg/ml tetracycline. Ki-67 expression was analyzed on day 6, 12, 15 and 21 after induction of differentiation by flow cytometry

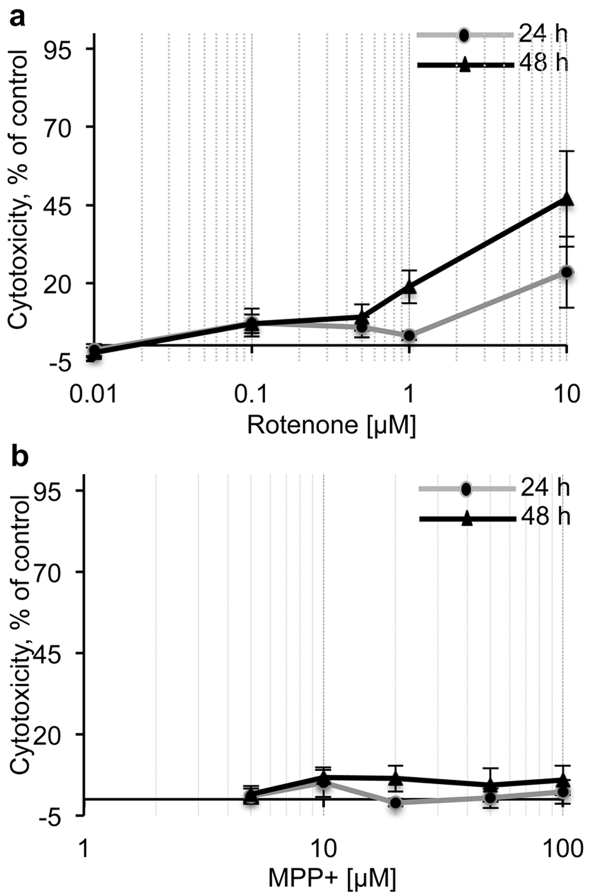


**Supplementary Figure S 2 Analysis of membrane integrity in LUHMES aggregates after exposure to rotenone and MPP$^+$.** LUHMES cells were differentiated following 3D+T10 protocol and exposed reversely to different rotenone (**a**) and MPP$^+$ (**b**) concentrations from day 6 to day 8 (48 h) and from day 7 to day 8 (24 h). Cytotoxicity was analyzed using LDH release assay and is presented in % of positive (1% Triton X100) controls in four independent experiments (n = 4, mean ± SEM)

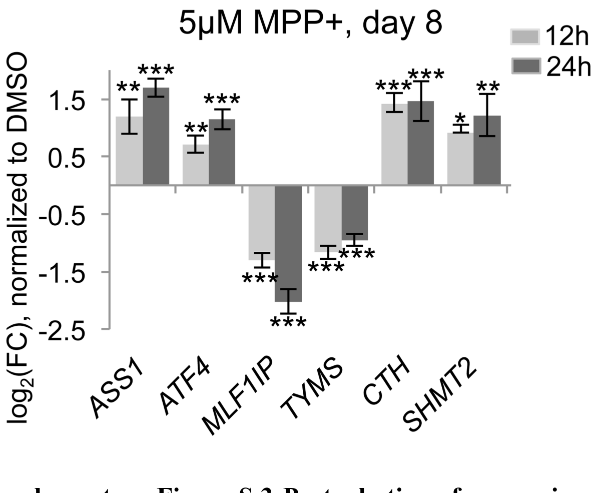


**Supplementary Figure S 3 Perturbation of expression of the genes involved in C1 metabolism, oxidative stress, and DNA repair by 5 µM MPP+.** ASS1, argininosuccinate synthase, AT4, activating transcription factor 4, CTH, cystathionase (cystathionine γ-lyase), MLF1IP, centromere protein U (MLF1 interacting protein), SHMT2,

Serine hydroxymethyl-transferase, and TYMS, thymidylate synthetase. The data are means of log2 (fold change) ± SEM of three independent experiments (9 technical replicates). ($n=3$, *$p<0.05$, **$p<0.01$, and ***$p<0.001$, one-way-ANOVA followed by Dunnett's post hoc test)
